# Taxonomic and functional analyses of intact microbial communities thriving in extreme, astrobiology-relevant, anoxic sites

**DOI:** 10.1186/s40168-020-00989-5

**Published:** 2021-02-18

**Authors:** Alexandra Kristin Bashir, Lisa Wink, Stefanie Duller, Petra Schwendner, Charles Cockell, Petra Rettberg, Alexander Mahnert, Kristina Beblo-Vranesevic, Maria Bohmeier, Elke Rabbow, Frederic Gaboyer, Frances Westall, Nicolas Walter, Patricia Cabezas, Laura Garcia-Descalzo, Felipe Gomez, Mustapha Malki, Ricardo Amils, Pascale Ehrenfreund, Euan Monaghan, Pauline Vannier, Viggo Marteinsson, Armin Erlacher, George Tanski, Jens Strauss, Mina Bashir, Andreas Riedo, Christine Moissl-Eichinger

**Affiliations:** 1 Diagnostic and Research Institute of Hygiene, Microbiology and Environmental Medicine, Medical University of Graz, Graz, Austria; 2 Department of Microbiology and Archaea Center, University of Regensburg, Regensburg, Germany; 3 UK Center for Astrobiology, School of Physics and Astronomy, University of Edinburgh, Edinburgh, UK; 4 Institute of Aerospace Medicine, Radiation Biology Department, German Aerospace Center (DLR), Cologne, Germany; 5 Centre de Biophysique Moléculaire, Centre National de la Recherché Scientifique (CNRS), Orléans, France; 6 European Science Foundation (ESF), Strasbourg, France; 7 Instituto Nacional de Técnica Aeroespacial – Centro de Astrobiología (INTA-CAB), Madrid, Spain; 8 Centro de Biología Molecular Severo Ochoa, Universidad Autónoma de Madrid (UAM), Madrid, Spain; 9 Leiden Observatory, Universiteit Leiden, Leiden, The Netherlands; 10 MATIS, Reykjavík, Iceland; 11 Faculty of Food Science and Nutrition, University of Iceland, Reykjavik, Iceland; 12 Institute of Environmental Biotechnology, Graz University of Technology, Graz, Austria; 13 Alfred Wegener Institute Helmholtz Centre for Polar and Marine Research, Periglacial Research Unit, Potsdam, Germany; 14 Division of Endocrinology and Metabolism, Department of Internal Medicine, Graz, Austria; 15 Sackler Laboratory for Astrophysics, Leiden Observatory, Leiden University, Leiden, The Netherlands; 16 BioTechMed, Graz, Austria

**Keywords:** Extreme environments, Microbiomes, Archaea, Bacteria, Propidium monoazide, Astrobiology, Space-analogue, Extremophiles, Extraterrestrial life, Metagenomics

## Abstract

**Background:**

Extreme terrestrial, analogue environments are widely used models to study the limits of life and to infer habitability of extraterrestrial settings. In contrast to Earth’s ecosystems, potential extraterrestrial biotopes are usually characterized by a lack of oxygen.

**Methods:**

In the MASE project (Mars Analogues for Space Exploration), we selected representative anoxic analogue environments (permafrost, salt-mine, acidic lake and river, sulfur springs) for the comprehensive analysis of their microbial communities. We assessed the microbiome profile of intact cells by propidium monoazide-based amplicon and shotgun metagenome sequencing, supplemented with an extensive cultivation effort.

**Results:**

The information retrieved from microbiome analyses on the intact microbial community thriving in the MASE sites, together with the isolation of 31 model microorganisms and successful binning of 15 high-quality genomes allowed us to observe principle pathways, which pinpoint specific microbial functions in the MASE sites compared to moderate environments. The microorganisms were characterized by an impressive machinery to withstand physical and chemical pressures. All levels of our analyses revealed the strong and omnipresent dependency of the microbial communities on complex organic matter. Moreover, we identified an extremotolerant cosmopolitan group of 34 poly-extremophiles thriving in all sites.

**Conclusions:**

Our results reveal the presence of a core microbiome and microbial taxonomic similarities between saline and acidic anoxic environments. Our work further emphasizes the importance of the environmental, terrestrial parameters for the functionality of a microbial community, but also reveals a high proportion of living microorganisms in extreme environments with a high adaptation potential within habitability borders.

**Video abstract**

**Supplementary Information:**

The online version contains supplementary material available at 10.1186/s40168-020-00989-5.

## Background

In order to understand the potential habitability of extraterrestrial environments, researchers analyse the physiological limits of (microbial) life, thriving in terrestrial, so-called analogue sites [1, 2]. These sites resemble extraterrestrial environments in one or several characteristics, and their biochemistry and biology can help to answer the question of whether life beyond Earth could exist, and if so, where and how it could be detected [3, 4]. Observations made from analogue sites directly feed into the design and preparation of life detection missions destined for Mars and elsewhere.

A large number of analogue environments has been investigated, from deserts like Atacama to deep subsurface environments [3, 5, 6] (additional references, see [1]). However, unavoidably in the majority of terrestrial settings, most of these environments are oxygenated and are thus inappropriate for studying conditions for extraterrestrial life, as most known extraterrestrial environments are oxygen-free or contain very low abundances of oxygen. For example, the thin Martian atmosphere contains 0.14% (v/v) oxygen and its surface and subsurface is therefore expected to be only habitable for microorganisms capable of tolerating or growing under an oxygen-free atmosphere [1].

Consequently, motivated by a desire to understand the characteristics of anaerobic life at its physical and chemical limits, the MASE project (Mars Analogues for Space Exploration; http://mase.esf.org/) was initiated, with the goal to use analogue sites as models for profound microbiome, chemical and instrumentation-based analyses [1] (additional details in Table 1 and Additional file 2: Supplementary Table 1).

**Table 1:** Overview on all sites and samples (additional information given in Additional file 2: Supplementary Table 1 and [1])

Sampling sites: types and locations		Sample names	Relevant chemical and physical extreme characteristics (example analogue Martian site)
Sulfidic springs (Germany)	Sippenauer Moor	**Sulfidic spring SM**; sulfidic spring SM^*^	Anoxic, low in organics, sulfur-rich, low temperature (10°C, Noachian sulfur cycling at Meridian Planum and across Noachian Mars)
	Islinger Mühlbach	Sulfidic spring IM; sulfidic spring IM^*^	
Hypersaline environment (Boulby mine, UK)	Sampling site 1	Hypersaline environment	Anoxic, low in organics, hypersaline (Terra Sirenium; anoxic brine slopes)
Acidic lake (Lake Grænavatn, Iceland)	Sampling site 1	Acidic lake SS1; acidic lake SS1^*^	Anoxic, low in organics, basaltic environment (acidic lake), acidic (pH 2–3), (water-rock interactions at Jezero Crater; Meridiani Planum)
	Sampling site 3	**Acidic lake SS3**; acidic lake SS3^*^	
Acidic river (Rio Tinto, Spain)	Lago Peligroso	Acidic river LP; acidic river LP^*^	
	Galdierias	Acidic river Gal; acidic river Gal^*^	
Permafrost setting (Herschel Island, Canada and Yedoma, Russia (SOB-14))	SlpD14-PS1-11	Permafrost SlpD14-1; permafrost SlpD14-1^*^	Low temperature (< 0°C, anoxic; Planum Boreum)
	SlpD14-PS3-11	Permafrost SlpD14-3; permafrost SlpD14-3^*^	
	SOB-14-06-A-37	**Permafrost SOB**; permafrost SOB^*^	
	TSD-14-IW1-01	Permafrost TSD; permafrost TSD^*^	
**Additional samples** (retrieved during Mars landing simulation campaign)			
Kaunertaler Glacier (Austria)	Sampling site 1	Glacier SS1; glacier SS1^*^	Low temperature (< 0 °C, Planum Boreum)
	Sampling site 2	Glacier SS2; glacier SS2^*^	

(^*^) in the sample abbreviations indicates pre-treatment of the samples with propidium monoazide (PMA). The sample “hypersaline environment” was not subjected to PMA treatment, as PMA treatment is inefficient in high-salt samples [7]. Samples used for shotgun metagenomics analyses are printed in bold

As none of all possible terrestrial analogue sites combines all extraterrestrial physical and chemical conditions at once [3], we chose to analyse a representative set of anoxic terrestrial environments with discrete physical and chemical parameters. We concentrated specifically on (i) low water activity (resembling, e.g. putative Martian recurring slope lineae brines [8–10]), (ii) low temperature (resembling, e.g. permafrost/ice deposit regions on current Mars/Planum Boreum), (iii) oxygen limitation/anoxic conditions (all sites), (iv) restricted availability of (complex and/or organic) nutrients (all sites) and (v) acidic conditions (resembling, e.g. Early Mars streamlets/water-rock interactions) (Table 1). With a combination of different methods, including large-scale cultivation and propidium monoazide [11] (PMA) amplicon/shotgun metagenome sequencing from 13 selected sites at five sampling locations (sulfidic springs, hypersaline environments, two acidic aquatic environments, permafrost settings), we characterized the bacterial and archaeal communities with respect to taxonomic composition and functional capabilities. Based on our results, we inferred general principles of anaerobic microbial communities in extreme, anoxic analogue terrestrial sites.

## Methods

### Sampling sites

Sediment, water and soil samples were obtained during sampling campaigns performed in 2014 and 2015. Sampling sites were selected based on their relevant chemical and physical characteristics (explained in detail in [1], Table 1, and Additional file 2: Table 1). In brief, the sampling sites included two sulfidic springs [12–17], (i) one subsurface, hypersaline environment [6, 18–20], (ii) one acidic lake [1, 21], (iii) one acidic river [22, 23] and (iv) two permafrost environments, later on referred to as the MASE environments. For comparison, one glacier environment was added, from which samples were retrieved during a Mars landing simulation of the Austrian Space Forum (ÖWF) in 2015 (AMADEE-15 [24], Additional file 1: Figure S1). Samples were taken with sterilized, DNA-free tools, transported under cooled conditions (< 10 °C), and processed as soon as possible. Samples from permafrost were kept frozen until processing. Samples for cultivation were taken as described in [1]; cultivation approaches, isolation of microorganisms and genome sequencing of representative isolates is described in the Additional file 1.

### PMA treatment and DNA extraction for shotgun metagenomics analyses and 16S rRNA gene amplicon sequencing

In order to discriminate between viable (i.e. cells with an intact cell membrane) and dead cells (cells with a disrupted cell membrane) in subsequent molecular analyses, 0.25 g of each sample was mixed with 1 ml of DNA-free H_2_O LiChrosolv® (Merck, USA) and treated with propidium monoazide (PMA; VWR, Austria) following the protocol of [25] as soon as possible after sampling. After adding a final concentration of 50 μM of PMA, the samples were briefly vortexed and gently shaken on ice for 10 min under dark conditions. After a light exposure time of 3 min (PMA-LITE Photolysis device; Biotium, USA), the samples were stored at − 80 °C until DNA extraction. The sample “hypersaline environment” was not subjected to PMA treatment, as PMA treatment is inefficient in high-salt samples [7].

In the following, all PMA-treated samples are marked with an asterisk (*), e.g. the sample “sulfidic spring IM*” refers to the PMA treated sample, whereas sample “sulfidic spring IM” refers to the PMA untreated, simultaneously processed sample. DNA extraction followed the standard operation procedures (SOPs) provided by the Earth microbiome project [26], by using the PowerSoil® DNA Isolation Kit (MOBIO Laboratories, Carlsbad; “Max” version for metagenomics analyses). Procedure controls (extraction blanks, etc.) were processed for each step of the analyses along with the samples.

### Generation of 16S rRNA gene amplicons, library preparation and Illumina MiSeq paired-end sequencing

The DNA concentration was normalized to 10 ng and used as a template in two distinct PCR reactions with primers carrying an Illumina MiSeq-compatible barcode adapter. The first reaction targeted bacterial and archaeal 16S rRNA genes (“universal” primer set [27]; forward primer F515, reverse primer R806). The second approach targeted Archaea exclusively and included a nested approach: In the first of the two subsequent PCR reactions, the template was amplified using the primer combination Arch344F (5′-ACGGGGYGCAGCAGGCGCGA-3′) and Arch915R (5′-GTGCTCCCCCGCCAATTCCT-3′ [28, 29]. In the second PCR, the amplicons for Illumina sequencing were generated by the tagged primers S-D-Arch-0349-a-S-17 and S-D-Arch-0519-a-A-16 [30] using the purified products (10 ng) of the first PCR as a template [31].

The cycling conditions for the universal approach were 94 °C: 3 min, 35 cycles of 94 °C: 45 s/60 °C: 60 s/72 °C: 90 s, and 72 °C: 10 min. For the first PCR of the nested PCR approach for Archaea, the cycling conditions were 95 °C: 2 min, 10 cycles of 96 °C: 30 s/60 °C: 30 s/72 °C: 60 s, 15 cycles of 94 °C: 30 s/60 °C: 30 s/72 °C: 60 s, and 72 °C: 10 min. For the second amplification, the cycling conditions were 95 °C: 5 min, followed by 25 cycles 95 °C: 40 s/63 °C: 120 s/72 °C: 60 s, and 72 °C: 10 min. Library preparation and sequencing was carried out at the Core Facility Molecular Biology at the Center for Medical Research, Medical University of Graz, Austria. Briefly, PCR products were normalized using a SequalPrep™ normalization plate (Invitrogen, USA). Each sample was indexed with a unique barcode sequence by an 8 cycles index PCR. Sequencing was carried out using the Illumina MiSeq device and MS-102-3003 MiSeq Reagent Kit v3-600 cycles (2 × 251 cycles).

### Community profiling based on amplicon sequencing

Reads from amplicons were processed using R (version 3.2.2) and the package DADA2 [32] as already described elsewhere [33], following the SOPs as recommended by the developers. Merged sequences were trimmed to a consistent length of ~ 270 bp (“universal” primer set) and ~ 140 bp (“Archaea” primer set). Thereafter, the sequences were assigned to a taxonomy using the RDP training set classifier v.14 and the SILVA v.123 classifier. Ribosomal sequence variants (RSVs) which were overlapping in negative controls and samples were removed from the datasets. All RSV tables are available in the Additional file 2 (Supplementary Tables 2-5). An additional data quality check was performed by visualization of rarefaction curves (richness vs. reads sampled), which confirmed sufficient sampling depth by reaching plateaus in each sample. Sequence data visualization of the amplicon data was carried out using the R package phyloseq [34]. The networks were created using the “make_network” function implemented in the phyloseq package with default parameters and additional settings given in the text.

### Shotgun library preparation and NGS for metagenomic analysis

Shotgun metagenomic analyses were performed on six selected samples (indicated in Table 1, in bold). One microgram of DNA was used for whole genome shotgun sequencing. Double-stranded DNA was quantified with the Qubit 2.0 (Invitrogen, USA). Shotgun libraries for Illumina MiSeq sequencing were prepared with the NEBNext® Ultra II DNA Library Prep Kit for Illumina® in combination with the Index Primers Set 1 (NEB, Germany) according to manufacturer’s instructions and as described in [35]. Briefly, 100–200 ng of dsDNA were fragmented by ultrasonication in a Bioruptor® instrument (Diagenode S.A., Belgium) with 4 cycles of 30 s on and 30 s off. The sheared DNA was used in end repair and adapter ligation reactions in the NEBNext® Ultra II DNA Library Prep Kit for Illumina® according to manufacturer’s instructions with size selection to an approximate inset size of 500–700 bp. Subsequent PCR amplification was performed in 4–6 cycles and libraries were eluted after successful amplification and purification in 33 μl 1× TE buffer pH 8.0. For quality control, libraries were analysed with a DNA High Sensitivity Kit on a 2100 Bioanalyzer system (Agilent Technologies, USA) and again quantified on a Quantus™ Fluorometer (Promega, Germany). An equimolar pool was sequenced on an Illumina MiSeq desktop sequencer (Illumina, USA). Libraries were diluted to 8–10 pM and run with 1% PhiX and v3 600 cycles chemistry according to manufacturer’s instructions on two MiSeq runs.

### Gene-centric data analysis of metagenomic reads

We used FastQC v. 0.11.5 [36] to determine the base quality throughout the 250 bp MiSeq-generated paired-end reads. Identified adapter and overrepresented homo-oligonucleotides were removed using cutadapt v 1.14 [37] and retained reads were further trimmed using Prinseqlite v. 0.20.4 [38] and following parameters: “-min_len 100 -trim_qual_right 20 -trim_qual_left 20 -trim_left 8”. BBMap short read aligner v. 37.61 was used to remove bacteriophage PhiX174 contaminants from trimmed high-quality reads by mapping them against the respective genome. Matching reads were not included in further analysis. Quality-filtered reads were then compared against the NCBI non redundant database using DIAMOND BLASTx v 0.9.10 [39] and default parameters.

Gene-centric analysis was performed through MG-RAST [40] and MEGAN [41]. Resulting taxa and functional gene tables were visualized and analysed using Calypso [42]. Datasets for comparative metagenomics were available through the public datasets of MG-RAST.

### Genome-centric analysis of metagenomic reads

Raw fastq files were quality filtered with trimmomatic 0.36 [43] based on fastqc 0.11.5 [36] file reports including removal of TrueSeq3 paired end adaptor sequences, truncating reads to a minimum length of 50 bp and a phred score of 20 in a sliding window of 5 bp. Quality-filtered reads were then assembled with Megahit [44] using the --presets meta-sensitive. Resulting final contigs and scaffolds were binned with MaxBin 2.2.4 [45]. Individual bins were evaluated including estimates for completeness and contamination with checkM. Taxonomic lineages of each bin were annotated with amphora2. Fifteen (of 122 bins) representative draft genomes with a mean level of completeness of 90% (cutoff min. 77%) and with a mean level of contamination of 10% (cutoff max. 20%) were further annotated and analyzed in MaGe [46] and replication rates were estimated with iRep [47] after mapping contigs on quality-filtered reads with Bowtie2 [48] and SAMtools [49]. All genome-centric analysis were supported and curated by a gene-centric approach including mappings of quality-filtered reads using blastX searches against NCBInr with diamond [39] and further analysis in MEGAN [50].

### Data availability

Sequence datasets obtained for microbial community data analysis were submitted to EBI and are publicly available (study project number: PRJEB18706). Shotgun datasets and binned genomes, as well as the genomes of the isolates *Buttiauxella* MASE-IM-9, *Yersinia* MASE-LG1, *Halanerobium* MASE-Boulby, are available through Bioproject number PRJEB28336.

## Results

### Samples and sampling sites

Overall, we retrieved samples from 13 selected sites at five sampling locations. All sampling locations were extreme sites, which were selected based on their astrobiology-relevant chemical and physical characteristics [1] (Table 1, Additional file 2: Supplementary Table 1).

All samples were subjected to microscopic examination, including fluorescence *in situ* hybridization (Additional file 1: Supplementary Fig. S2), cultivation (see Additional file 1) and DNA-based analyses.

### The intact archaeal communities differ between the sites and reflect niche association

Twenty-five samples from six different sites were subjected to PMA treatment and subsequent microbiome analysis (designated with “*”, Table 1). PMA was used to mask background DNA from dead cell material [11].

In a first step, we focused on the archaeal community. In total, 787,842 archaeal reads were obtained using the Archaea-targeting primer set, resulting in 1,502 archaeal RSVs. On average, 37,516 reads were obtained from each sample, the number of retrieved reads varied strongly across the samples. The lowest number of sequences was retrieved from sample “acidic lake SS3” (72 reads), whereas the highest read count was obtained for “sulfidic spring IM*” (PMA-treated sample of sulfidic spring Islinger Muehlbach; 106,622 reads). It should be noted that no archaeal RSVs were observed from the samples obtained from the glacier (PMA treated and untreated), which indicates either a low amount of archaeal 16S rRNA genes (below detection limit) or an insufficient primer match [51, 52]. A bar chart displaying the archaeal community composition (phylum level) of all samples (PMA treated and untreated) is given in Additional file 1: Supplementary Figure S3.

**Fig. 1:**
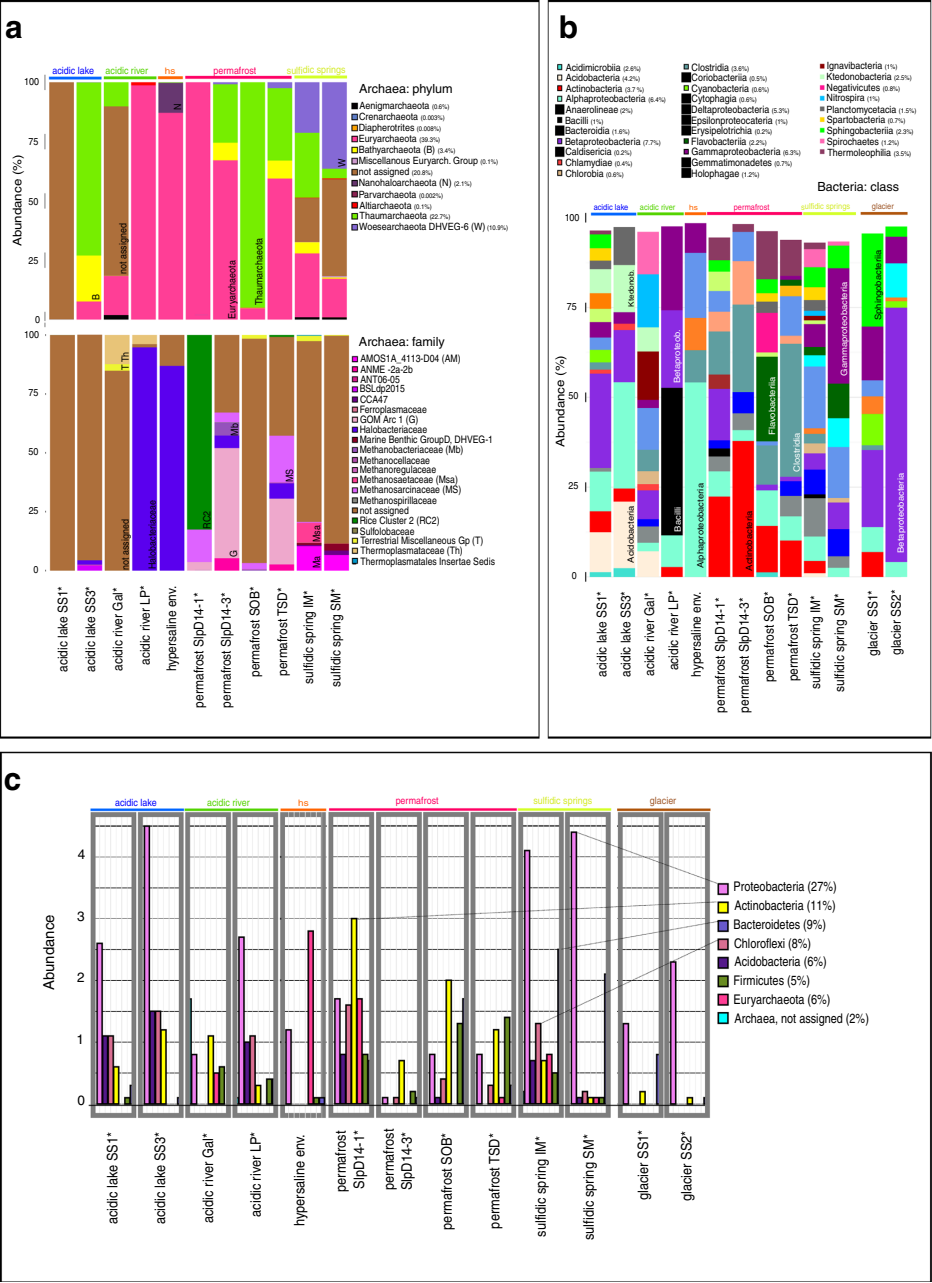
Archaeal and bacterial profiles of intact microbial communities from MASE environments. Panel **a** shows the taxonomic profile obtained using the Archaea-targeting primer, on phylum (upper bar chart) and family level (below). Phyla names are followed by relative abundances (all datasets); no archaeal signatures were obtained from the glacier samples. Panel **b** shows the bacterial composition of all samples on class level (only those classes with > 0.1% are shown). Panel **c** displays the most abundant phyla of the microbial community (based on “universal” primer set, “microbiome”). For all panels, the relative abundance of each taxon is shown on the *y*-axis. The total relative abundance, summed up for all samples, is given in brackets behind the taxa names in the legends. For unclassified RSVs (“unassigned”) the highest assigned taxonomic level is given. For instance, “Archaea, not assigned” reflects all RSVs which were classified on Archaea level, but could not be assigned to other taxonomic levels. Full detail on the archaeal and universal dataset is given in Additional file 2: Supplementary Tables 2 and 3.

In the PMA dataset, which included the PMA-untreated hypersaline environment sample [7], the majority of RSV counts were assigned to the phyla Euryarchaeota (39.3%), Thaumarchaeota (22.7%), unassigned Archaea (20.8%) and Woesearchaeota (DHVEG-6; 10.9 %; Fig. 1a). Signatures of Euryarchaeota were detected throughout all Archaea-positive samples, except for the acidic lake SS1* sample. The highest proportion of haloarchaeal signatures was observed in the sample of the hypersaline environment (Halobacteriales; 99% of all Euryarchaeota signatures within this sample). Other sequences from the same sample were classified as Nanohaloarchaeota (12.6% of all archaeal sequences), Thaumarchaeota and Woesearchaeota (both below 1%).

Notably, 99.9% of the sequences detected in the acidic lake SS1* remained unclassified within the archaeal domain and, thus, might represent a new taxon, whereas all sequences of the second sampling site of the acidic lake SS3* could be assigned on phylum level and were classified as potentially methane-producing Bathyarchaeota [53, 54] (19.4% of all sequence counts within this sample), potentially ammonia-oxidizing Thaumarchaeota [55] (72.9%), Euryarchaeota (7.6%) and a minor proportion of Woesearchaeota (DHVEG-6; < 1%).

Sequences from Rio Tinto were mainly assigned to unclassified Archaea (71.4%), Thaumarchaeota (10.1%) and Euryarchaeota (16.5%; in the acidic river Gal* sample) and a high proportion of Euryarchaeota (98.8% in the acidic river LP* sample).

In the spring biotopes, a remarkably high proportion of signatures assigned to Woesearchaeota (DHVEG-6) was detected (36.5% and 21.2% of all sequence counts in SM* and IM* samples, respectively), in addition to Altiarchaeales [14, 15] and other signatures. Notably, only the sulfidic spring biotope revealed signatures of Archaea belonging to the group of Aenigmarchaeota (< 1%).

Permafrost samples contained mainly signatures of Euryarchaeota with high proportions of methanogenic archaea (*Methanobacteria* and *Methanomicrobia*) and Thaumarchaeota (Soil Crenarchaeotic Group; SCG and Marine Benthic Group; MBG). Notably, the “universal primer” approach (see next section) revealed a slightly different composition of the archaeome (see Additional file 1: Supplementary Information Figure S4).

### The microbiomes of MASE sites harbour a vast diversity of adapted, anaerobic microorganisms

In a next step, we amplified both 16S rRNA gene pools of PMA treated and PMA-untreated samples with a “universal” primer set to target the entire microbial community. In total, we obtained 1,523,276 sequences (minimum: 10,819, “permafrost SlpD14-3*”; maximum: 119,379, “acidic river Gal*”; mean: 60,851). After processing the reads, a total of 15,945 different RSVs were obtained. In the following, we concentrate on the intact microbial proportion (PMA-treated samples, and the PMA-untreated “hypersaline environment”); the taxonomic profile of all PMA-untreated samples is given in Additional file 1: Supplementary Information Figure S5.

Intact microbial communities in MASE environments were generally characterized by Proteobacteria (27 %, mean percentages of total amount of RSV counts), followed by Actinobacteria (11 %), Bacteroidetes (9 %), Chloroflexi (8 %), Acidobacteria (6 %), Firmicutes (5 %), Euryarchaeota (6 %) and a minor proportion (2 %) of unassigned Archaea (Fig. 1b, c). Signatures of Proteobacteria were present throughout all sampling sites, but in different proportions. Similarly, signatures of Actinobacteria were detected in each environment with the exception of the “hypersaline environment”. This biotope also revealed a minor proportion of Firmicutes signatures, which were completely absent in samples from the acidic lake* environment, but were present in all other samples.

In the datasets, we identified the signatures of a broad diversity of facultatively or obligately anaerobic and extremotolerant/extremophilic microorganisms with relevance for analogue research and astrobiology [56–58]. Those included methanogens (e.g. *Methanobacterium*), halophiles (e.g. archaeal Halobacteria, Halothiobacillaceae), acidophiles (e.g. Acidithiobacillaceae) and a number of taxa associated with psychrotolerance (e.g. *Pseudomonas*, *Oscillatoria*, *Phormidium* and *Arthrobacter*; Additional file 2: Supplementary Table 2). In addition, we identified signatures of taxa with relevant anaerobic metabolic capabilities [59–61], including iron reduction (e.g. *Geobacter*, and *Shewanella*), sulfate reduction (e.g. *Desulfobacterium*, *Desulfovibrio*), nitrate-dependent iron oxidation (e.g. *Aquabacterium*, *Thermomonas*) and methanogenesis (e.g. *Methanobacterium*, *Methanosaeta*, *Methanoperedens*).

As of potential interest for planetary protection measures for space missions [62], it should be mentioned that signatures from potentially radiation and/or desiccation-resistant microbes such as members of the Deinococcus-Thermus phylum (*Deinococcus*, *Truepera* and additional unclassified members) were also detected in the glacier*, sulfidic spring*, permafrost* and acidic river* samples. Additionally, signatures of spore-formers, such as bacilli and Clostridia, generally capable of resisting harsh environments, were found in all environments.

### The MASE sites reveal microbiome-based similarities which are shaped by the extreme environmental parameters

The highest archaeal richness and diversity (Archaea-targeted approach, “archaeome”) was observed in the sulfidic spring biotopes (PMA-treated samples: observed species: 598 (mean), Shannon index: 5.13 (mean); PMA-untreated samples: observed species: 461 (mean), Shannon index: 4.79 (mean); Additional file 2: Supplementary Table 6), whereas samples from the acidic lake (pH 3) contained an overall very low archaeal richness and diversity (PMA-treated samples: observed species: 20 (mean), Shannon index: 0.81 (mean); PMA-untreated samples: observed species: 9 (mean), Shannon index: 1.80 (mean)). With respect to archaeal richness and diversity, PMA-treated and -untreated samples (all sites) did not differ significantly (Additional file 2: Supplementary Table 6).

**Fig. 2:**
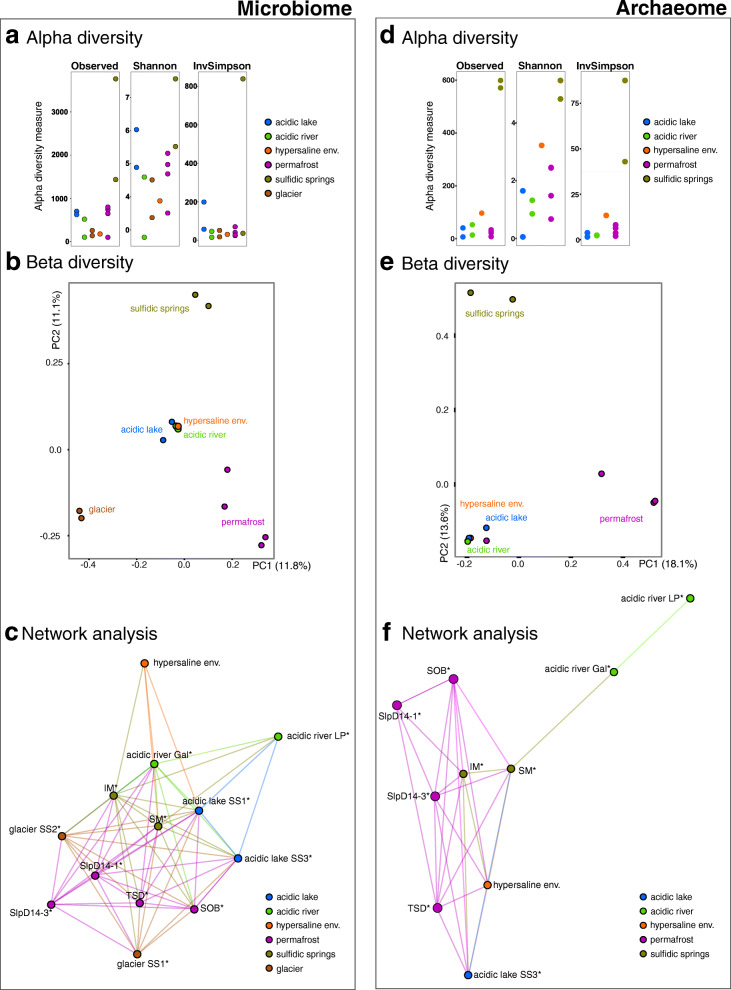
Microbial diversity and network analysis of microbial communities in samples from MASE sites (only PMA treated samples shown). The left panel gives information on the general microbiome (universal dataset), whereas the right panel displays results from the archaeome analyses (Archaea-targeted approach). The alpha diversity (**a**, **d**), beta diversity (PCoA plots; unweighted Bray-Curtis distance; **b**, **e**) and the connections between in the sampling sites (networks; **c**, **f**) are displayed separately. The connection (based on Bray-Curtis similarity) of samples is visualized by the number of straight lines in-between, reflecting a distance of 0.4 or below (see text)

The samples from sulfidic springs also showed the highest diversity when observing all microbial signatures (universal dataset, “microbiome”; PMA-treated samples: observed species: 2589 (mean), Shannon index: 6.51 (mean); PMA-untreated samples: observed species: 2438 (mean), Shannon index: 6.28 (mean); Additional file 2: Supplementary Table 7), whereas the lowest diversity was observed in samples from the hypersaline environment (observed species: 155, Shannon index: 3.8). Alpha diversity information of all PMA-treated samples is displayed in Fig. 2.

The lowest proportion of RSVs from intact cells was detected in samples from permafrost (15.3%, sample SLpD14-3; 64%, sample TSD), acidic lake (30%, sample SS1) and the acidic river (70%, sample LP; 80%, sample Gal; Additional file 2: Supplementary Table 7, column “observed”).

The beta diversities of the archaeome and the general microbiome were visualized via PCoA plots (based on Bray-Curtis distances; Fig. 2b, e). The microbial communities derived from the acidic environments, such as the acidic river and acidic lake (although obtained from geographically remote sampling sites in Iceland and Spain), clustered in both PCoA plots (Fig. 2b, e), along with the sample from the “hypersaline environment” (UK). The permafrost samples (with one exception in the archaeome analysis), the sulfidic spring samples and the glacier samples grouped separately into their own clusters.

To analyze the microbial order of connectivity amongst sampling sites, networks based on archaeome and microbiome were constructed (thresholds: max. distance 1.0, line weight 0.4; Fig. 2c, f). Regarding the archaeome, the samples derived from acidic environments (acidic lake and acidic river) were not highly connected to other sampling sites (only one connecting edge; Fig. 2f). The permafrost samples showed a high level of connection to the sulfidic springs and one acidic lake sample. The microbiome network revealed similar connections with a relatively central position of the sulfidic springs, which also carried the highest microbiome diversity (Fig. 2f, a). Notably, the microbiome from the hypersaline environment, representing an environment with dissimilar physical and chemical parameters, was connected primarily with environmental samples from the acidic sites. It appears that the high ionic strength in these sites is reflected by a more similar microbiome composition. This observation was confirmed by the detection of twenty overlapping taxa, of which eight were resolved on genus level, namely *Paludibacter*, *Aquabacterium*, *Geobacter*, *Sulfurovum*, *Beggiatoa*, *Thiothrix*, *Spirochaeta_2* and *Opitutus*. Notably, Halobacteriaceae were found in both environments, as well as Acidobacteriales. Most of the core genera of the hypersaline environment and acidic sites were, however, affiliated to Proteobacteria (Additional file 1: Supplementary Information Figure 6).

Fitting environmental variables, such as water activity, temperature and pH values, onto the NMDS ordinations revealed that the microbial community variation might be best explained by temperature (Additional file 1: Supplementary Information Figure 7), followed by pH values. Water activity measurements were similar in each site (mean water activity value *a*_w_ = 0.95), except for the “hypersaline environment”, which had the lowest water activity value (*a*_w_ = 0.75).

In a next step, we were interested in whether the MASE sites share certain microbial signatures. The intact core microbiome of all sampling sites (PMA-treated samples and hypersaline environment) comprised four taxa. Two of them could be classified on the genus level, namely *Paludibacter* (12 different RSVs present in all sampling sites, 0.3% of all RSV counts) and *Opitutus* (34 different RSVs present in all sampling sites; 0.1% of all RSV counts). At the species level, *Paludibacter propionicigenes*, an obligately anaerobic bacterium producing propionate originally isolated from a rice plant residue [63], was closely related to one RSV from the sulfidic spring SM*, whereas type strain *Opitutus terrae*, a strictly anaerobic, also propionate (and acetate) producing microorganism isolated from a rice paddy soil microcosm [64] was represented by two RSVs from the acidic river Gal* and permafrost* (Additional file 1: Supplementary Information Figure 8a).

In a second step, we excluded the PMA-untreated hypersaline environmental sample with the result that the intact core microbiome of all remaining MASE sites exhibited 34 common taxa (Additional file 1: Supplementary Information Figure 8b, c). Seven of them were resolved at the genus level (*Bryobacter* (35 different RSVs), Candidatus *Solibacter* (22), *Acidocella* (9), *Bdellovibrio* (34), *Aquicella* (16), *Opitutus* (34) and *Paludibacter* (14); Additional file 1: Supplementary Information Figure 8b), and 20 common taxa could be resolved on the order level (Additional file 1: Supplementary Information Figure 9). Most RSVs belong to the family Anaerolinacaea (239 different RSVs), followed by members of the order Gaiellales (92) and the family Planctomycetaceae (88). Notably, we could not detect any archaeal core taxa on any taxonomic level. RSVs assigned to *Paludibacter* and members of the family Opitutacea were also identified in the shotgun metagenomics dataset (in PMA and PMA untreated sulfidic spring sample, abundance < 0.1%; see below).

### PMA treatment affects the abundance of Eukaryota in the metagenomics datasets

Samples from three representative sampling sites (i.e. sulfidic spring SM, acidic lake SS3 and permafrost SOB) were selected for metagenomic sequencing. For each sample, we processed two parallel fractions, one PMA treated, the other untreated. The other samples of the MASE sites could not be included in the metagenomic approach, as obtained DNA concentrations were too low for a proper analysis.

Overall, the taxonomic information derived from the metagenomics dataset (Additional file 2: Supplementary Table 8) was congruent with the findings from amplicon sequencing approach. However, the permafrost sample SOB showed a higher contribution of Acidobacteria and the archaeal TACK superphylum (i.e. Thaumarchaeota), when PMA treatment of the sample was performed.

It shall also be noted that the signatures of Alveolata and Amoebozoa (Eukarya) were found to be reduced after PMA treatment in the acidic lake samples, indicating a possibly high contribution of free DNA therefrom (Additional file 1: Supplementary Fig. 10).

The highest taxonomic diversity was detected in SM samples and least in the permafrost SOB samples (*p* = 0.047, ANOVA, based on Shannon Index). Metagenomic signatures of *Bradyrhizobium*, Mycobacteriaceae and uncultured microorganisms were found in all PMA-treated samples from all locations (“PMA core”), whereas the overall core microbiome was predominated by unclassified microbial signatures, *Streptomyces*, *Mycobacterium*, unclassified PVC-group organisms, unclassified Proteobacteria and *Bradyrhizobium* (Additional file 2: Supplementary Table 8).

### The functional profiles of the MASE microbial communities mirror the extreme physical and chemical characteristics of the MASE sites

As mentioned above, metagenomic analyses could only be performed for three representative MASE sites (each with and without PMA). However, for the sake of completeness, we performed comparative analysis of all samples using the in silico tool Tax4fun [65] to infer the estimated microbial functions from amplicon-based information. The methodology and results of this approach are provided in Additional file 1: Supplementary Figure 11 and its legend.

In the metagenomics dataset, the diversity of microbial functions was found to be significantly higher (Shannon index, *p* = 0.027, ANOVA) in the permafrost sample (SOB), compared to SM and SS3, where it was found to be the lowest diversity. While the grouping of microbial functions according to location was found to be significant (*p* = 0.01, redundancy analysis), the grouping according to PMA treatment was not found to be significant.

**Fig. 3:**
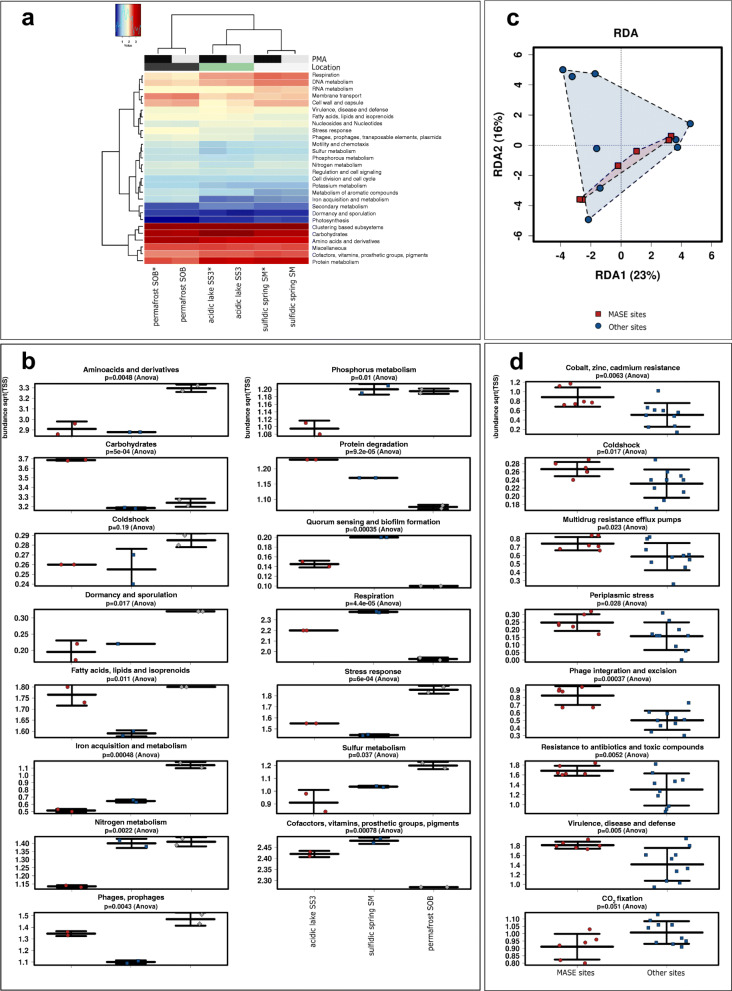
Metagenomics-based, functional profiles of selected MASE sites (sulfidic spring SM, acidic lake SS3 and permafrost SOB). Overview on the found functional pathways and functions and their relative abundance (**a**), and the difference in the functional profiles of the different MASE sites (**b**). The comparison with other, non-extreme, but comparable sites (soil, groundwater, lake) does not reveal significant impact on the metagenomics profile (**c**). MASE site differ significantly from other moderate (soil, groundwater, lake) or extreme (brine, Antarctic lake, permafrost) environments in a number of functions (**d**)

The most abundant enzymes/functions detected in the shotgun metagenome dataset were (i) an integrase (involved in phage integration and excision), (ii) glycosyltransferase (biosynthesis of galactoglacans and related lipopolysaccharides), (iii) cation efflux system protein CusA (cobalt zinc cadmium resistance), (iv) long-chain fatty acid CoA ligase (fatty acid degradation regulons) and (v) decarboxylase (serine glyoxylate cycle) (additional information is given in Fig. 3 and Additional file 2: Supplementary Table 9).

The metagenome-derived microbial functions of the three environments showed differences in the levels of certain metabolic pathways and functions. In particular, the permafrost sample SOB was characterized by an increased turnover of amino acids, increased cold shock response, dormancy/sporulation, iron acquisition and metabolism, stress response and sulfur metabolism, reflecting a potential tension induced by diurnal and seasonal freeze thaw cycles due to environmental temperature changes. The sulfur spring SM was particularly characterized by quorum sensing and biofilm formation, respiration, co-factor formation and, together with permafrost sample SOB, nitrogen and phosphorus metabolism. The acidic lake sample SS3 was characterized by the highest level of functions involved in carbohydrate turnover and protein degradation and, together with sample SOB, in fatty acid metabolism (Fig. 3a, b).

In a following step, we wanted to understand the differences between MASE microbial communities in taxonomic and functional composition when compared to metagenomic datasets from moderate environments, namely soil (Hungary, Finland, South Africa), groundwater (Tulsa, USA), lake- (Switzerland, Greece) and seawater (Mediterranean Sea, Indian Ocean). For comparison, we also included other extreme environments, namely brines (Spain), Antarctic lake (Antarctica) and permafrost (Axel Heiberg Island, Canada) (Additional file 2: Supplementary Table 9). Amongst all these environments, the permafrost samples were characterized by the lowest taxonomic diversity (assessed on genus level, Shannon index, *p* = 0.0016, ANOVA), whereas the samples from lakes, groundwater and sulfidic spring showed the highest diversity. When we compared MASE sites-associated taxa with the non-extreme, but comparable sites (soil, groundwater, lake), the samples still grouped according to their sample type and MASE samples did not significantly separate from the non-extreme samples (Fig. 3c).

In a next step, we compared the microbial functions from MASE sites with all other sites mentioned above. The MASE environments revealed significantly higher levels in, e.g. cobalt, zinc and cadmium resistance, functions involved in cold-shock, multidrug resistance/efflux pumps, periplasmic stress, phage-integration and excision, resistance to antibiotics and toxic compounds, virulence disease and defense (Fig. 3d), indicating that indeed the selected MASE sites are more extreme. Notably, a substantially lower level of functions involved in CO_2_ fixation (*p* = 0.051) was observed for the MASE sites, reflecting a reduced capacity for autotrophy (Fig. 3d).

### The omnipresence of organics could impair analogue studies

In the course of metagenomics analyses, we reconstructed a number of distinct draft genomes from the PMA treated and PMA-untreated sample sets. Overall, 15 bins of appropriate quality were obtained (70% completeness: max. 5% contamination, 80% completeness: max. 10% contamination, 90% completeness: max 20% contamination), which are summarized in Table 2.

**Table 2:** Overview on the obtained binned genomes, their origin, taxonomy and details on completeness and contamination

Origin	Sample/bin	Taxonomy	Details (completeness %/ contamination %)
Sulfidic spring	1Sipp, bin 2	Gammaproteobacteria, Thiotrichales	97.87/19.98
	1Sipp, bin 3	Gammaproteobacteria, Chromatiales	88.68/4.45
	1Sipp, bin 6	Gammaproteobacteria, Chromatiales	80.70/ 7.21
	1Sipp, bin 9	Epsilonproteobacteria, *Sulfurovum*	79.86/12.80
	1Sipp, bin 5	Gammaproteobacteria	79.88/10.50
	PMA 2Sipp, bin 2	Bacteroidetes, *Chlorobium*	90.86/8.66
Icelandic lake	3Ice, bin 1	Alphaproteobacteria, *Acidiphilium*	99.49/3.83
	3Ice, bin 5	Planctomycetes, Planctomycetaceae	97.73/12.65
	3Ice, bin 7	Actinobacteria, *Acidimicrobium ferooxidans*	84.48/15.83
	PMA 4Ice, bin 5	Unknown bacterium	96.30/19.97
	PMA 4Ice, bin 4	Acidobacteria, *Acidobacterium capsulatum*	95.85/6.47
	PMA 4Ice, bin 20	Bacteroidetes	80.77/6.68
Permafrost	5SOB, bin1	Gammaproteobacteria, *Pseudomonas* sp.	98.70/0.94
	5SOB, bin3	Bacteroidetes, *Flavobacterium*	84.36/15.26
	PMA 6SOB, bin1	Gammaproteobacteria, *Pseudomonas* sp.	98.70/0.76

Within the core genome of all 15 retrieved genome bins, 46 gene families and 2897 genes were identified. These were, amongst others, prophage and phage-related functions, pilus/flagella formation, detoxification, adaptation to stress, ferrous iron uptake, phosphorus metabolism and, again, heterotrophic lifestyle (amino-acids, sugar/mannose metabolism, protein degradation), confirming the observation that MASE-associated microorganisms are adapted to extreme environments, but prone to heterotrophic life style. Notably, intactness and activity of the MASE-associated microorganisms were also supported by calculations of their replication rates with iRep. This software uses the coverage information of high quality MAGs to determine copy numbers between the origin of replication and the terminus to define iRep values. These values can be further interpreted as a trade-off between the proportion of the population involved and the number of replication events (further details on interpreting iRep values are available at [66]). According to this analysis, at least one representative draft genome was actively replicating per MASE site. The highest activity (iRep 2.05, could refer to 25–100% of the population was actively replicating 1–4 times) was observed for *Acidimicrobium ferooxidans* from the Icelandic lake.

The predisposition for heterotrophic life styles were further confirmed by the core functions of the retrieved isolates (see Additional file 1: Supplementary Information for more details on the cultivation approach; isolates are listed in Additional file 2: Supplementary Table 10; list of core functions is given in Additional file 2: Supplementary Table 11), as the majority of isolates obtained throughout the entire study showed a heterotrophic lifestyle (despite the attempt to grow autotrophs as well). When we predicted and compared the functional capacity of the isolates and the core microbiome in silico (Additional file 1: Supplementary Information Figure 12), we recognized a rather selective enrichment of particularly heterotrophic microorganisms in the cultivation approach that was used. Additionally, the core genome of the three model isolates (details also in the Additional file 1: Supplementary Information; 34 genes overlapping), revealed, besides ribosomal proteins, elongation factors and regulators, a number of cold-shock and stress-involved genes (such as *cspA*, *cspl*, *scpD* or *cspC*), indicating the necessity to adapt to extreme environments.

Based on all our analyses, we can confirm that the MASE sites are indeed extreme environments for the microorganisms, but also the central role of organic molecule metabolism became obvious. Recognizing this central principle, we determined the total organic carbon (TOC) of the MASE samples, which ranged from 0.12% (acidic lake) up to 22.7% (permafrost SOB; Additional file 1: Supplementary Figure 13). These values were largely in agreement with literature values from comparable sites [67]. Although the acidic lake was found to contain the lowest amount of TOC, the associated microbial functions showed the highest specialization in carbohydrate turnover, whereas the microbial community in the permafrost SOB sample showed the highest abundance for amino acid-associated functions.

## Discussion

Organisms in extreme anoxic environments on Earth, and more speculatively on other planetary bodies, if it exists, would have to cope with diverse physical and chemical stressors. These include low water availability (desiccation, high salinity), extreme temperatures, high and low pressures, nutrient and oxygen limitations and radiation [68]. In order to assess habitability for an extraterrestrial environment, researchers aim to understand the chemical and physical boundaries of life in general. For this, they analyze suitable model organisms, including bacteria (e.g. *Hydrogenothermus marinus*, a desiccation tolerant bacterium able to grow in the presence of perchlorates [69]), Archaea (e.g. methanogens [70] or haloarchaea [71]) and fungi [72], but also natural microbial communities thriving in analogue biotopes [5, 73].

Planetary analogues are selected on the basis of geology, mineralogy, topography and environmental conditions, depending on the planet or body of interest, such as Mars or the Icy Moons. One of the most obvious model ecosystems are permafrost environments [58], deserts and high-salt biotopes, as well as Antarctic and subsurface environments [3]. It has been discussed that these analogue environments are incapable of combining all chemical and physical characteristics of interest; however, these environments were considered good models to analyze the impact of one or a few extreme conditions on the biology therein.

In this study, we performed one further step in optimizing research for Mars-analogue sites, by the exclusion of oxygenated model environments. Samples from selected analogue sites were collected under anoxic conditions, and the living microbial community thriving therein was analyzed by a broad combination of anaerobic cultivation and molecular methodology to address the following questions: Which Bacteria and Archaea are alive under the extreme, anoxic, environmental conditions? Which functions do they possess? Can we cultivate them? Is there a core microbiome of all sites? Are there general principles of anaerobic microbial communities with impact on the habitability assessment and the search for life on Mars and beyond?

The retrieved data were intended to optimize the definition of habitable extraterrestrial environments and to deliver important information for future life detection missions.

To our knowledge, our study is the first that uses propidium monoazide (PMA) to mask background DNA from dead microorganisms in Mars-analogue extreme settings. This method allowed us to retrieve information on a vast diversity of the microbiomes and archaeomes of the intact and thus probably living microbial communities. Overall, the lowest proportion of intact (presumably alive) species signatures was obtained from permafrost samples and samples from the acidic environments (Additional file 2: Supplementary Table 7). The high amount of disrupted material therein was confirmed also through metagenomics analysis, and indicated by the high TOC value (high amount of microbial debris). Thus, the permafrost environment appeared to be one of the most extreme MASE biotopes.

Notably, we identified stringent similarities of the taxonomic profiles of microbial communities thriving in acidic and saline MASE environments (Fig. 2). This is particularly interesting, as acidic and saline environmental parameters are a likely combination for, e.g. Hesperian Mars settings [74].

The cultivation assay implemented in the MASE project comprised 1131 enrichment attempts, that resulted in 69 stable enrichments and 30 anaerobic, pure bacterial isolates and one archaeon (*Methanomethylovorans* sp.), all already or are currently being made available for the scientific community through the German culture collection DSMZ. It is our intention to exploit those model organisms and to analyze their physiological properties to understand their resistances to, e.g. radiation and to estimate, whether they could survive extraterrestrial conditions (see also [75–78]). Information on a diverse set of (anaerobic) taxa, which were present in all sampling sites could be retrieved, and also several signatures of known taxa with astrobiological relevance were detected [23, 79–82].

A cosmopolitan group of (mostly) mixotrophic and anaerobic microorganisms was able to reside in all MASE sites, amongst them *Opitutus* and *Paludibacter* [63, 64]. Notably, the co-existence of *Opitutus* and *Paludibacter* taxa has been already observed earlier, e.g. involved in anaerobic cycling of carbon in permafrost samples [83]. Both genera, however, are considered to be specialized on complex organic compound degradation [84].

In general, the MASE core microorganisms are of great interest, since they could represent excellent model organisms for studying adaptation and resistance properties. Their obviously very flexible lifestyle, combined with specific resistance and adaptation capacities, could allow them to adapt quickly and thus to follow chemical and physical evolution of a certain environment.

The general, functional principles of all MASE microbial communities were two-fold: on the one hand, microbial signatures were characterized by resilience and resistance against different characteristics potentially representing extraterrestrial stressors such as (metal) ionic strength (increased cobalt, zinc, and cadmium resistances) and, e.g. freezing (increased cold shock-involved functions, Fig. 3).

On the other hand, all levels of our analysis indicated a strong dependency of the terrestrial microbial communities on complex organic matter, in both moderate and extreme environments, as indicated, e.g. by a lowered CO_2_ fixation capacity in MASE sites (Fig. 3).

Even if we, in our study, ruled out the terrestrial oxygen contamination in order to match extraterrestrial conditions as much as possible, the analogue sites were characterized by the (terrestrial) omnipresence of organic compounds, shaping the microbial communities substantially.

## Conclusions and outlook

Our study has contributed novel insights into the microbiology of analogue sites. In particular, a number of highly valuable model organisms has been retrieved, which directly feeds into the other goals of the MASE project, namely studying the limits of growth of selected isolates, deciphering the molecular principles of resistances [78], analysing the genomic and metabolomic inventory of representative microbes [75], studying the fossilization processes and detectability of biomarkers during artificial fossilization [85] and the optimization of automated life detection [1, 86]. However, numerous tasks remain to be accomplished in future. These include (i) a comprehensive re-evaluation of the potential impact of the terrestrial organic load on the biology of analogue environments for space research, (ii) the extension of the dataset with additional microbiome data from other extreme environments, (iii) further testing of the hypothesis that a core microbiome in extreme anoxic environment exists, (iv) further identification of so-far unknown microbial taxa found in our molecular survey (v) and the improvement of (targeted) cultivation strategies to increase the available culture collection of microorganisms thriving in extreme, astrobiology-relevant terrestrial sites.

## Supplementary Information

**Additional file 1: Supplementary Figure 1**: Analogue astronauts in the field during the AMADEE-15 Mars-landing simulation mission. **Supplementary Figure 2**: CLSM micrographs of FISH from sulfidic spring samples. **Supplementary Figure 3**: Archaeal signatures obtained using the Archaea-targeting primer set. **Supplementary Figure 4**: Archaeal signatures obtained with the universal primer set. **Supplementary Figure 5**: Bacterial signatures obtained with the “universal” primer set, PMA untreated samples. **Supplementary Figure 6**: The core taxa of the hypersaline environment and the acidic environment*. **Supplementary Figure 7**: Parameters shaping the community. **Supplementary Figure 8**: The MASE core microbial taxa. **Supplementary Figure 9**: The core microbiome of PMA untreated samples depicted in a Venn diagram.**Supplementary Figure 10**: Phylum-level, taxonomic information derived from the metagenomic dataset. **Supplementary Figure S11**: Predicted KEGG3 pathways (Tax4fun) and network of functional genes of the MASE site microbiomes. **Supplementary Figure 12**: Comparison of metabolic pathways for the core microbiome and the cultivated microorganisms. **Supplementary Figure 13**: Total organic carbon in MASE samples, in %.

**Additional file 2: Supplementary Table 1**: Description of the MASE sampling sites. **Supplementary Table 2**: RSV table resulted by the "universal" primer approach. **Supplementary Table 3**: RSV table resulted by the "Archaea" primer approach. **Supplementary Table 4**: RSV table resulted by the "universal" primer approach (including negative controls). **Supplementary Table 5**: RSV resulted by the "Archaea" primer approach (including negative controls). **Supplementary Table 6**: Diversity calculations based on the archaea-targeted approach for each sampling site and biotope. **Supplementary Table 7**: Diversity calculations based on the microbiome data (universal dataset) for each sampling site and environment. **Supplementary Table 8**: Bacterial, archaeal and eukaryotic taxonomic information derived from the metagenomics dataset. **Supplementary Table 9**: Comparison with metagenomic datasets from non-MASE sites. **Supplementary table 10**: Comprehensive information on isolates obtained within the frame of the MASE project and their respective environmental source. **Supplementary Table 11**: Core genome of retrieved genome bins.

## Data Availability

The datasets generated and/or analyzed during the current study are either included as supplementary information/figure/table, or deposited at EMBL ENA, project numbers PRJEB18706 and PRJEB28336.
