# Supplementary material for: Taxonomic and functional analyses of intact microbial communities thriving in extreme, astrobiology-relevant, anoxic sites
Source: Microbiome. 2021 Feb 18;9:50. doi: 10.1186/s40168-020-00989-5 (PMC7893877; doi:10.1186/s40168-020-00989-5)
Supplement: Supplementary file 2 — Additional file 1: Supplementary Figure 1: Analogue astronauts in the field during the AMADEE-15 Mars-landing simulation mission. Supplementary Figure 2: CLSM micrographs of FISH from sulfidic spring samples. Supplementary Figure 3: Archaeal signatures obtained using the Archaea-targeting primer set. Supplementary Figure 4: Archaeal signatures obtained with the universal primer set. Supplementary Figure 5: Bacterial signatures obtained with the “universal” primer set, PMA untreated samples. Supplementary Figure 6: The core taxa of the hypersaline environment and the acidic environment*. Supplementary Figure 7: Parameters shaping the community. Supplementary Figure 8: The MASE core microbial taxa. Supplementary Figure 9: The core microbiome of PMA untreated samples depicted in a Venn diagram.Supplementary Figure 10: Phylum-level, taxonomic information derived from the metagenomic dataset. Supplementary Figure S11: Predicted KEGG3 pathways (Tax4fun) and network of functional genes of the MASE site microbiomes. Supplementary Figure 12: Comparison of metabolic pathways for the core microbiome and the cultivated microorganisms. Supplementary Figure 13: Total organic carbon in MASE samples, in %. [file 40168_2020_989_MOESM2_ESM.docx]

**Taxonomic and functional analyses of microbial communities thriving in extreme, astrobiology-relevant sites**

Alexandra Kristin Bashir^1,2^, Lisa Wink^1^, Stefanie Duller^1^, Petra Schwendner^3^, Charles Cockell^3^, Petra Rettberg^4^, Alexander Mahnert^1^, Kristina Beblo-Vranesevic^4^, Maria Bohmeier^4^, Elke Rabbow^4^, Frederic Gaboyer^5^, Frances Westall^5^, Nicolas Walter^6^, Patricia Cabezas^6^, Laura Garcia-Descalzo^7^, Felipe Gomez^7^, Mustapha Malki^8^, Ricardo Amils^8^, Pascale Ehrenfreund^9^, Euan Monaghan^9^, Pauline Vannier^10^, Viggo Marteinsson^10,11^, Armin Erlacher^12^, George Tanski^13^, Jens Strauss ^13^, Mina Bashir^14^, Andreas Riedo^15^, and Christine Moissl-Eichinger^1,16,*^

**Supplementary information**

Email: [christine.moissl-eichinger@medunigraz.at](mailto:christine.moissl-eichinger@medunigraz.at)

This file contains:

1. Information on the cultivation, 16S rRNA gene and genome sequencing of isolates
2. Information on the genomic analyses of selected MASE isolates
3. All supplementary Figures (Supplementary Figure 1- 13)
4. References

#### Cultivation, 16S rRNA gene and genome sequencing of isolates

**Methods**: As described in [1], microbial enrichments were retrieved using an anoxic, basic minimal medium (MASE I and MASE II). This basal medium was supplemented with a wide range of electron donors/electron acceptors to address different types of metabolic specialists, including autotrophs, heterotrophs, methanogens, iron reducers and oxidisers, sulfate-reducers, acidophiles and halophiles. Overall, 31 isolates (classified to genus level) were subsequently purified using various techniques such as the optical tweezer technology [2,3], anoxic plating or dilution series in liquid medium. 16S rRNA genes of the isolates were retrieved by Sanger sequencing of a full-length amplicon. The sequences were classified using EZBioCloud [4] and confirmed using the Alignment, Classification and Tree Service platform of Silva ([5]. Obtained isolates have and are currently being archived and will be available for the public at the Leibniz Institute DMSZ, the German Collection of Microorganisms and Cell Cultures.

**Results: Sixty-nine microbial enrichments, 31 isolates and three model organisms were obtained from the MASE sites**

Prior to cultivation, all samples retrieved were subjected to microscopic examination and fluorescence *in situ* hybridization. More details are given in the Supplementary Information, Figure S2.

Our cultivation efforts focused on anaerobic (i) fermenting microbes, (ii) sulphate reducers, (iii) C1 utilizers, (iv) nitrate reducers, (v) methanogens, (vi) polycyclic aromatic hydrocarbon (PAH) degraders, (vii) amino acid utilizers and (viii) iron reducers (a comprehensive description can be found in [1]). In total, we performed 1,131 enrichment attempts with varying supplements and were able to obtain 69 stable, microbial enrichments.

Despite our efforts to cultivate also autotrophic microorganisms, or organisms growing under astrobiology-relevant conditions, main growth was observed in heterotrophic enrichment attempts (10.4 % positive enrichments), and only in 1.9 % of enrichments targeting autotrophs. Overall, 31 pure cultures could be obtained (Table 2; Supplementary Table 6). A number of the pure cultures are meanwhile available through the German culture collection DSMZ (DSM numbers given in Supplementary Table 6). Thirty isolates belonged to the domain of the Bacteria (mostly Proteobacteria), and one isolate was affiliated with the domain of Archaea (*Methanomethylovorans sp.* MASE-SM-1). All of them grew under anoxic conditions and added organic medium supplements. Three bacterial isolates were selected for further genomic analyses, serving as model organisms for subsequent experiments [6,7], namely: *Yersinia intermedia* MASE-LG-1 (DSM 102845) [6,7], *Halanaerobium* sp. (MASE-BB-1; DSM 105537) [7], the only isolate from the hypersaline environment, and *Buttiauxella* sp. (MASE-IM-9; DSM 105071) from the sulfidic spring Islinger Muehlbach.

#### Genomic analyses of selected MASE isolates

**Methods**: Selected isolates were subjected to PacBio sequencing, performed at the DNA Sequencing and Genomics Laboratory at the University of Helsinki, Finland, which also provided the assembled and quality filtered reads. Whole genome analysis was performed using MaGe [8].

**Results**: *Halanaerobium* MASE-BB-1 (total genome length: 2.75 Mbp, 2,582 CDS, completeness: 99.13 %, GC content: 34.4%, protein coding density: 88.18 %, four identical copies of the 16S rRNA gene): The full length 16S rRNA gene was found to be 99.37 % identical to *Halanerobium fermentans* R-9 (typestrain). *Halanaerobium* sp. MASE-BB-1 was found to have full metabolic pathways for ethanol, alanine, aspartate, glutamine, cysteine, serine threonine and trypotophane degradation, cellulose degradation II (fungi), D-mannose, galactose, L-arabinan, L-rhamnose, lactose, maltose, melibiose, ribose, sucrose, trehalose, xylose. Beyond these fermentative capacities, there were indications that the C1 compound formaldehyde (formaldehyde oxidation V) can be used.

*Yersinia intermedia* MASE-LG1 (total genome length: 5.0 Mbp, 4,633 CDS, completeness: 99.63 %, GC content: 47.6 %, protein coding density: 83.98 %, seven identical (<0.0046 dissimilarity) copies of the 16S rRNA gene): The full length 16S rRNA gene was found to be 99.04 % identical with the type strain of *Y. intermedia* (ATCC 29909). *Yersinia intermedia* MASE-LG-1 was found to have full metabolic pathways for aerobactin synthesis (siderophore), glycerol, L-lactaldehyde, methylgloxal, allantoin, ethnanolamine, urea degradation, as well as for the degradation of numerous amino acids and carbohydrates (D-allose, D-mannose, fructose, glycogen, homogalacturonan, L-arabinose, L-lyxose, L-rhamnose, L-sorbose, lactose, melibiose, ribose, sucrose, trehalose, xylose). Indications for dissimilatory nitrate reduction were detected, as were the potential for fatty acid beta-oxidation. Arginine and glutamate-dependent acid resistance potential was found, as also the capacity of arsenate detoxification and superoxide radical degradation.

*Buttiauxella* sp. MASE-IM-9 (total genome length: 5.4 Mbp, 4,988 CDS, completeness: 100 %, GC content: 50.4 %, protein coding density: 87.64 %, eight identical (<0.004445 dissimilarity) copies of the 16S rRNA gene): The closest related type strains identified were *Buttiauxella ferragutiae* (ATCC 51602(T)) and *Buttiauxella agrestis* (ATCC 33320(T)) with 99.93 % 16S rRNA gene sequence similarity. Strain MASE-IM-9 showed a certain resistance potential, including antibiotic efflux systems, antibiotic inactivation potential (beta-lactamase/D-alanine carboxypeptidase) and genes involved in antibiotic target alteration (e.g. undecaprenyl phosphate-L-Ara4FN transferase). Similar to *Yersinia intermedia* MASE-LG1, *Buttiauxella* sp. possessed the pathway for siderophore biosynthesis (enterobactin); moreover, the metabolic pathways for (R,R)-butanediol, glycerol, L-lactaldehyde, methylgloxal degradation, as well as a variety of (poly-)amines and amino acid degradation pathways were found. It showed the capacity for aromatic compound (catechol, protocatechuate), and carbohydrate degradation ((1,4)-b-xylan, chitobiose, D-mannose, fructose, galactose, glycogen, homogalacturonan etc). It also possessed arginine and glutamate-dependent acid resistance potential, as also the capacity of arsenate detoxification and superoxide radical degradation.

#### Supplementary Figures

**Supplementary Figure 1: Analogue astronauts in the field during the AMADEE-15 Mars-landing simulation mission**. During this activity of the Austrian Space Forum (ÖWF) in 2015 [9], samples from the Kaunertal glacier were retrieved under simulation conditions.


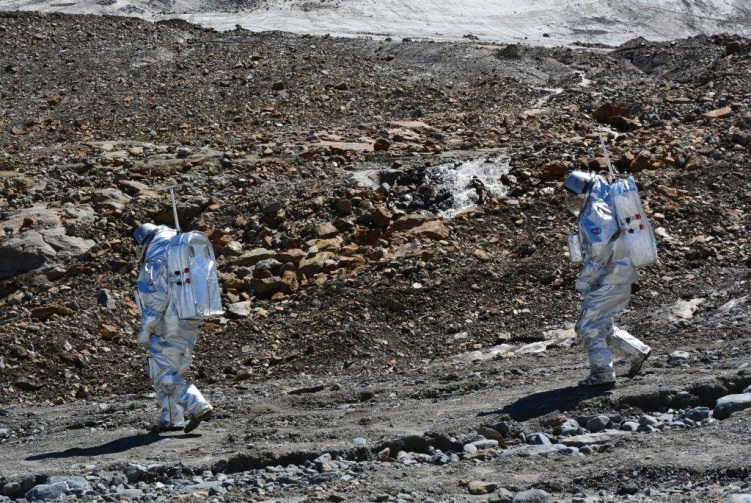


**Supplementary Figure 2: CLSM micrographs of FISH from sulfidic spring samples (IM (upper panel) and SM (panel below)) pinpoints to high abundance of Bacteria compared to Archaea.** Bacteria are labelled red and Archaea are labelled in green. In both samples a substantially higher amount of bacteria-positive cells is visible. The SM image shows additionally abiotic debris of unknown chemistry (whitish structures).

With fluorescence *in situ* hybridization clear microbial signals were obtained from sulfidic spring samples. Bacterial cells of diverse morphologies (cocci, rods, filaments) were identified, outnumbering the mainly coccoid archaeal cells. However, due to the low amount of microbial cells and numerous particles present in the sediment samples, the identification of microbial cell structures proved difficult.

For FISH, samples were fixed on site in paraformaldehyde (3 % (w/v), final concentration) and FISH was carried out as described earlier [10,11] using Archaea-specific probes (‘Arch-Mix’; Arch 344, Arch 915, Arch 1060; rhodamine green labelled) and EUB338/I; directed against Bacteria; CY3 labelled), 20 % (v/v) formamide and 0.01 % (w/v) SDS. DAPI was used as counterstain. A NONEUB probe[12] was used as nonsense negative control. The fluorescent dyes Cy3 (532 nm), rhodamine green (635 nm) and DAPI (488 nm) were sequentially induced. Additional auto- fluorescence was captured using a 405 nm laser beam.

Micrographs (confocal stacks) were acquired with a Leica ACS APO40x oil CS objective lens (NA, 1.15) and a Leica ACS APO 63x oil CS objective lens (NA, 30). Sample size depending appropriate numbers of optical slices were generated using a Z-step ranging from 0.2 - 0.7 μm. Imaris 7.3 (Bitplane, Switzerland) was used for volume visualization and post-process rendering of the micrographs. FISH samples in general exhibited a high amount of (soil) particles, which were visible using all of the three filters and hampered the distinction between cells and particles.


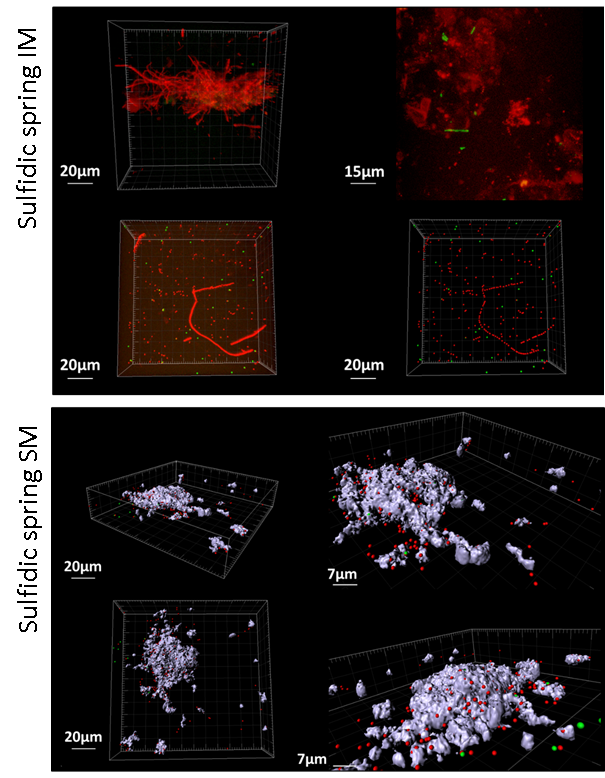


**Supplementary Figure 3: Archaeal signatures obtained using the Archaea-targeting primer set.** Taxonomic profile of archaeal signatures derived from PMA treated samples and their PMA untreated counterparts are shown on phyla level. The symbol * highlights PMA treatment.


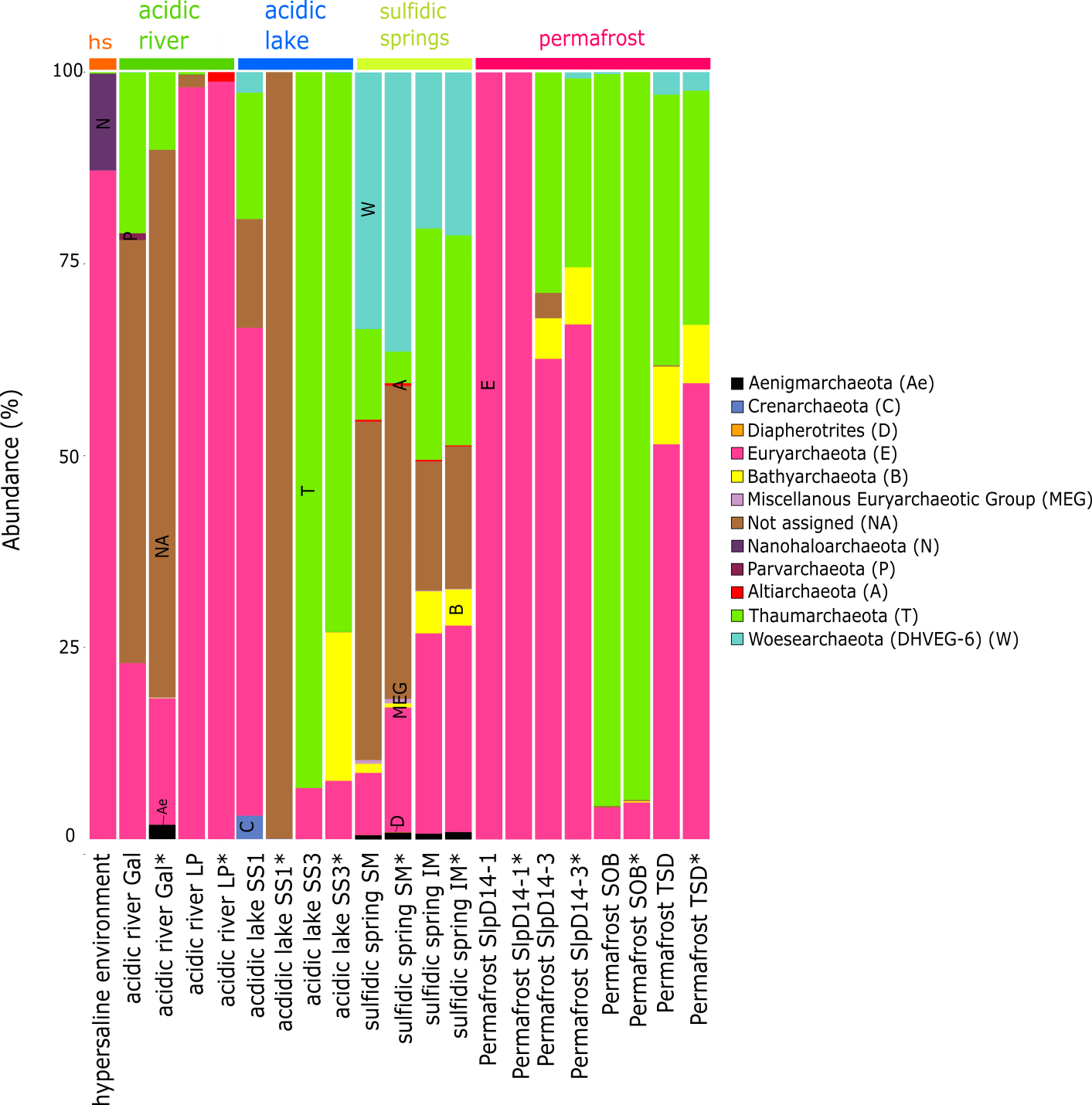


**Supplementary Figure 4: Archaeal signatures obtained with the universal primer set.** Taxonomic profile of the archaeal signatures derived of PMA treated samples and their PMA untreated counterparts are shown on class level. The symbol * highlights PMA treatment. Notably, no archaeal signal could be obtained for glacier samples except for the “glacier SS2*” sample. “hs” refers to hypersaline environment.


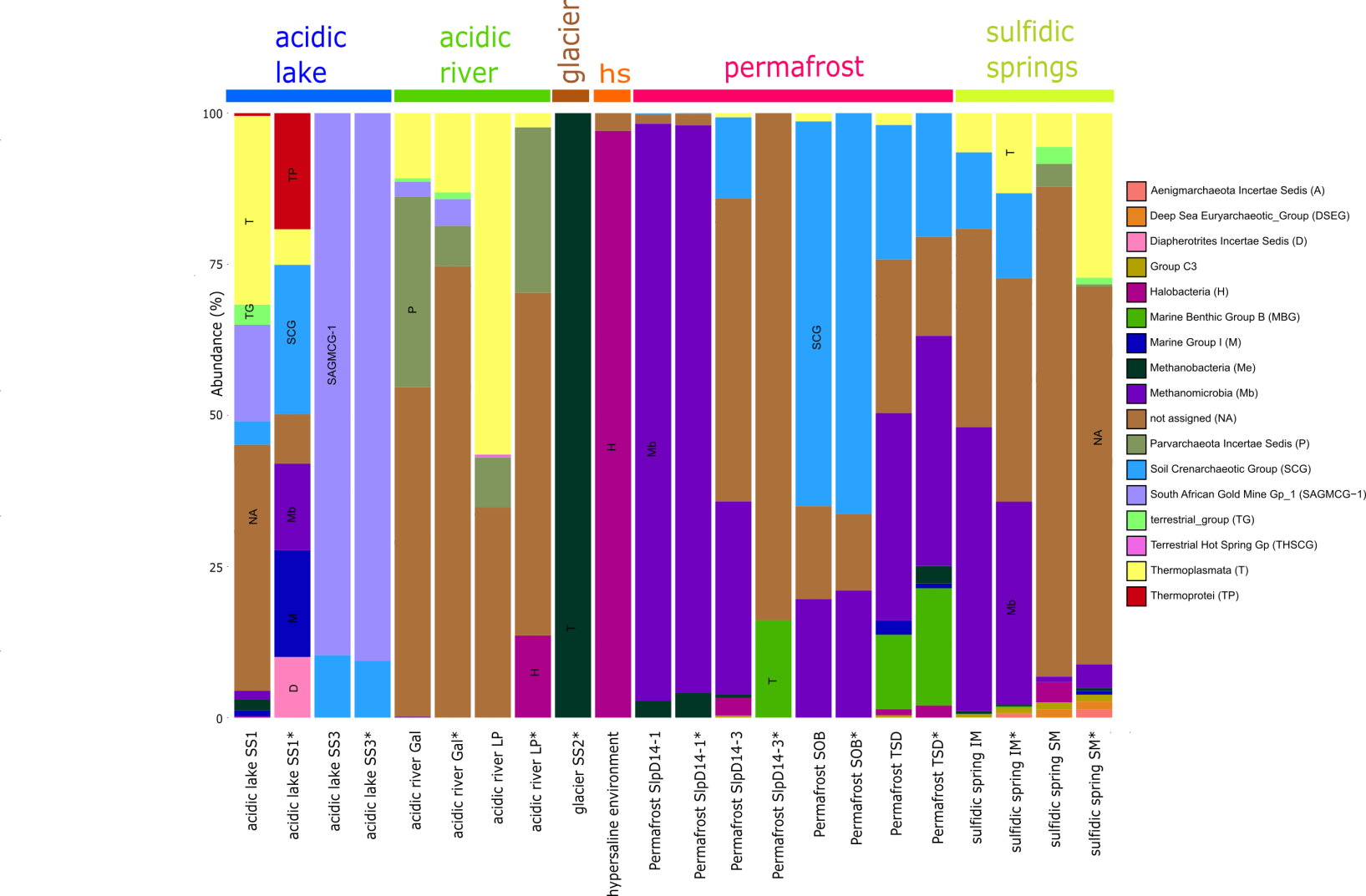


**Supplementary Figure 5: Bacterial signatures obtained with the “universal” primer set, PMA untreated samples.** Taxonomic profile of bacterial signatures of PMA treated samples and their PMA untreated counterparts are shown on class level. The symbol * highlights PMA treatment. The taxonomic profile of the “hypersaline environment” can be found in Fig. 1.


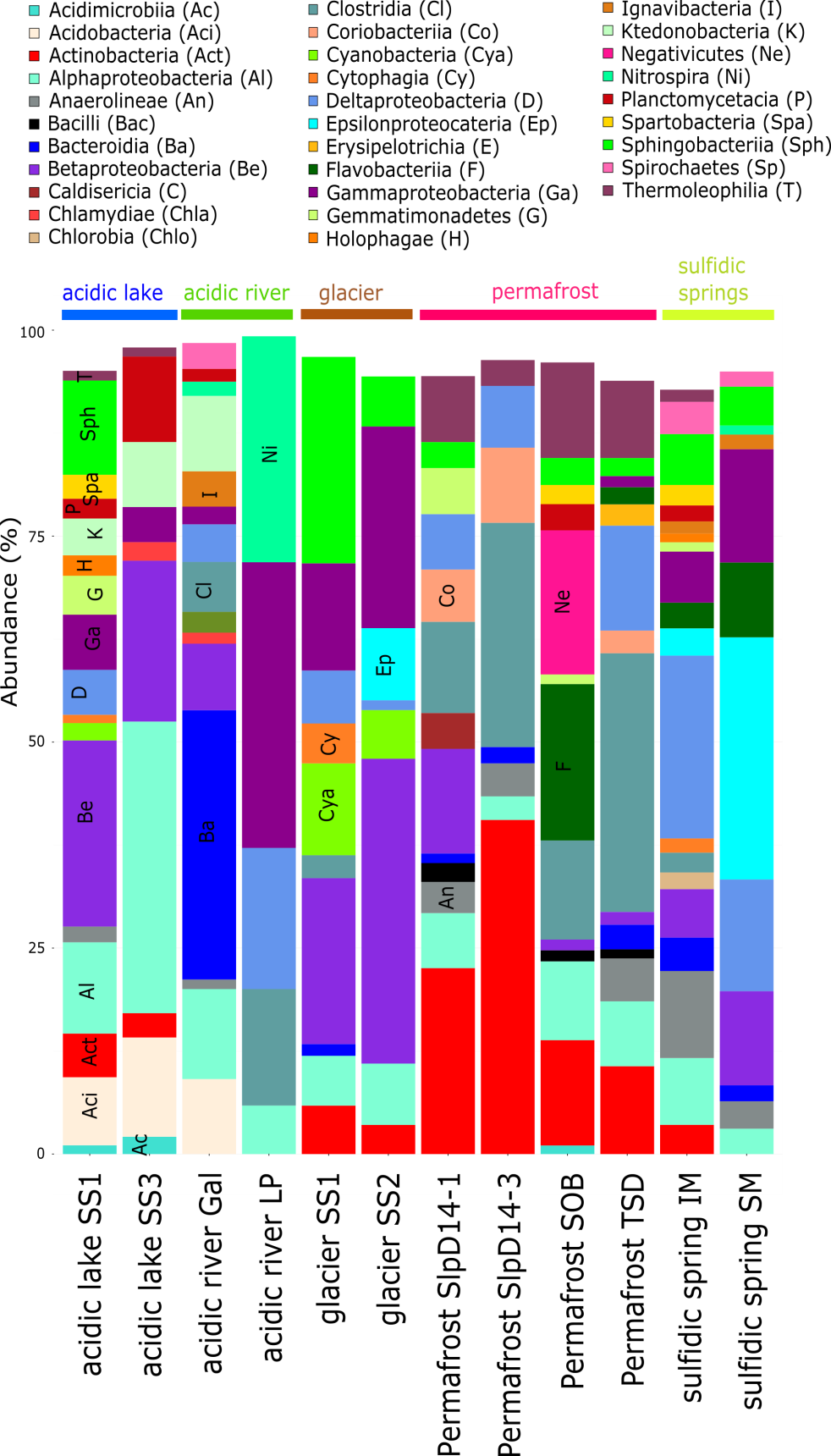


**Supplementary Figure 6: The core taxa of the hypersaline environment and the acidic environment* consists mainly of Proteobacteria.** Each lineage is resolved to the maximally assigned taxonomic level and each node represents an assigned taxonomic level. Circles segment taxonomic levels.


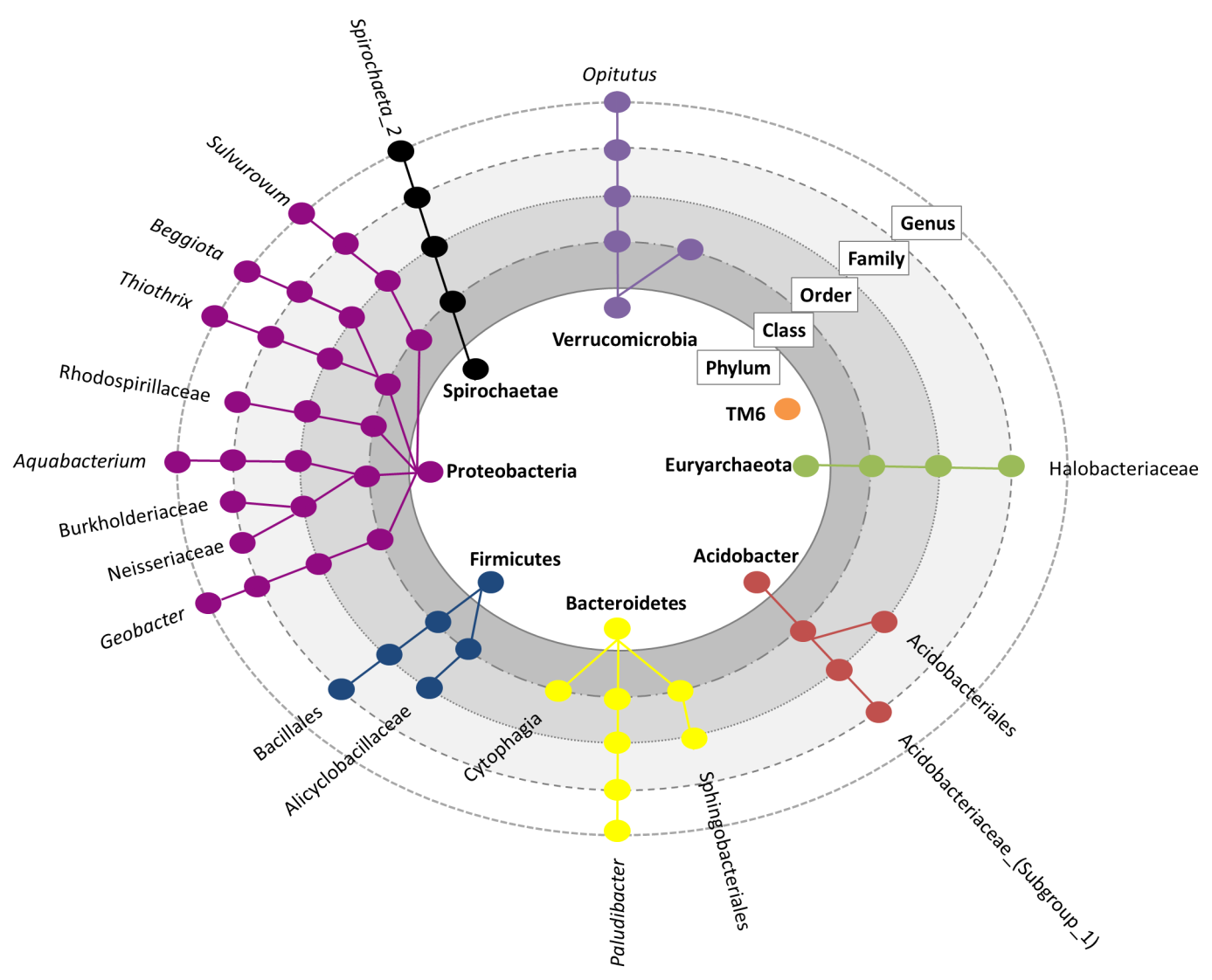


**Supplementary Figure 7: Parameters shaping the community.** The NMDS plots were run with 999 permutations (stress: 0.11). The left panel shows a low water activity is shaping the microbial community of the “hypersaline environment”. The highest influence on the microbial composition is given by the temperature as shown in the middle. The right panel displays the pH and it reveals that the low pH value in acidic environments is highly influencing the composition.

Water activity was determined using a Rotronic HP23-AW water activity meter (Rotronic, Germany). Prior to the measurement the meter was calibrated to five points (aw = 0.325, 0.595, 0.755, 0.845, and 0.935) using saturated calibration standards (MgCl_2_, NH_4_NO_3_, NaCl, KCl, and KH_2_PO_4_, respectively) prepared as described elsewhere [13]. Ten ml of each sample was measured. This process was repeated three times.


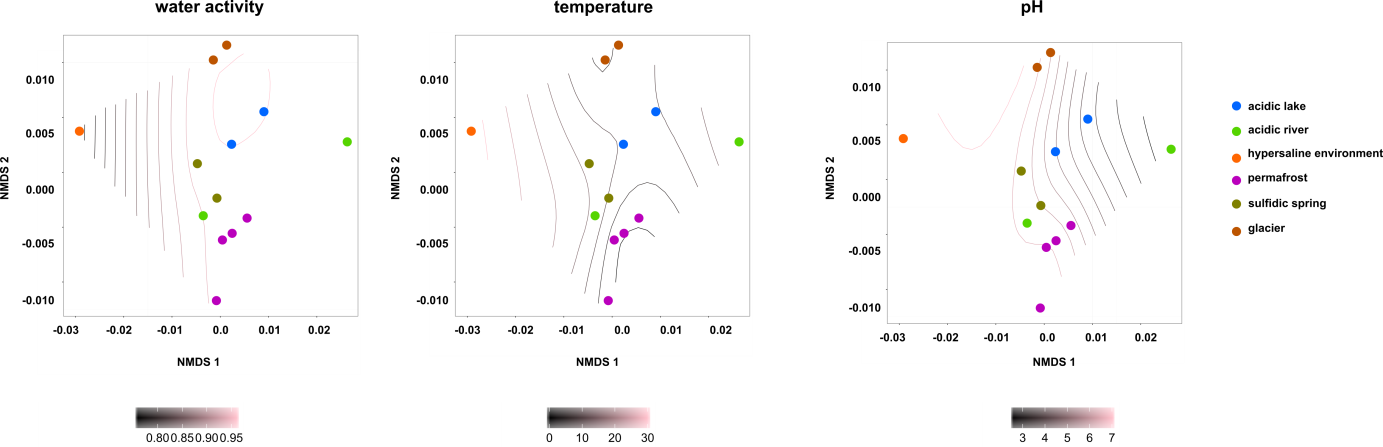


**Supplementary Figure 8: The MASE core microbial taxa.** (a) Venn diagram including all genera of samples treated with PMA and the PMA untreated hypersaline environment. Four genera were present in all samples. (b) and (c) display the core microbiome information, retrieved from all sampling sites except the hypersaline environment. The phylogenetic tree in (b) displays all RSVs, including sequence data information retrieved from corresponding available type strains (full taxonomic names given). (c) Venn diagram of the PMA core microbiome.

The 16S rRNA gene-based phylogenetic tree was calculated applying the Maximum-Likelihood algorithm, using the MEGA7 software [14]. For this, RSVs deriving from intact/viable organisms, which were common in all sampling sites and classifiable at least to class level, were used to generate an alignment with ClustalW [15]. The phylogenetic distances were calculated based on the Maximum-Likelihood approach (Jukes-Cantor model). The resulting tree was visualized using the web-based tool iTOL [16]. The Venn diagrams were created using the online tool InteractiVenn [17].


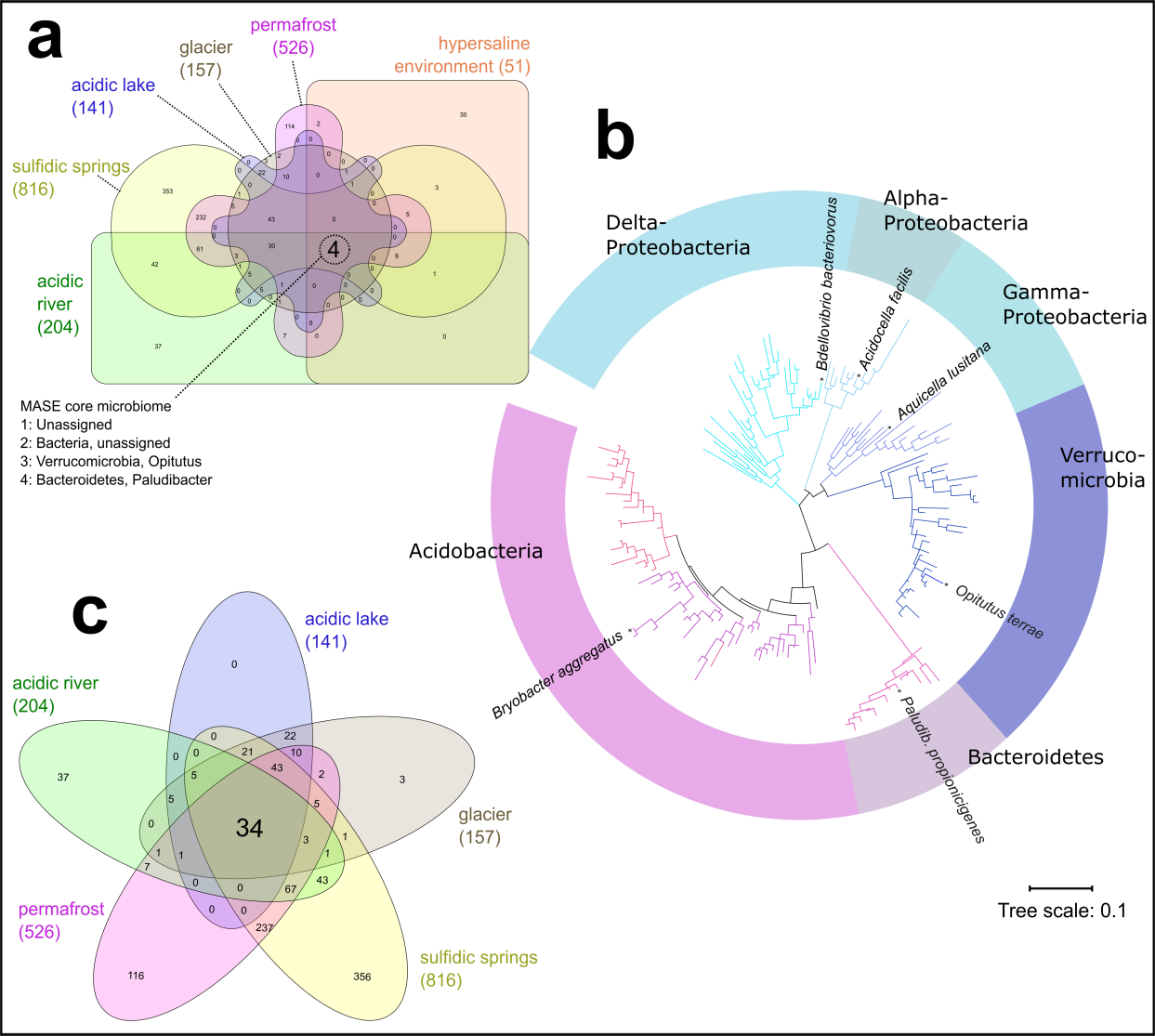


**Supplementary Figure 9: The core microbiome of PMA untreated samples depicted in a Venn diagram.** 20 core genera comprise the core genera. Most of them belong to unassigned Proteobacteria.


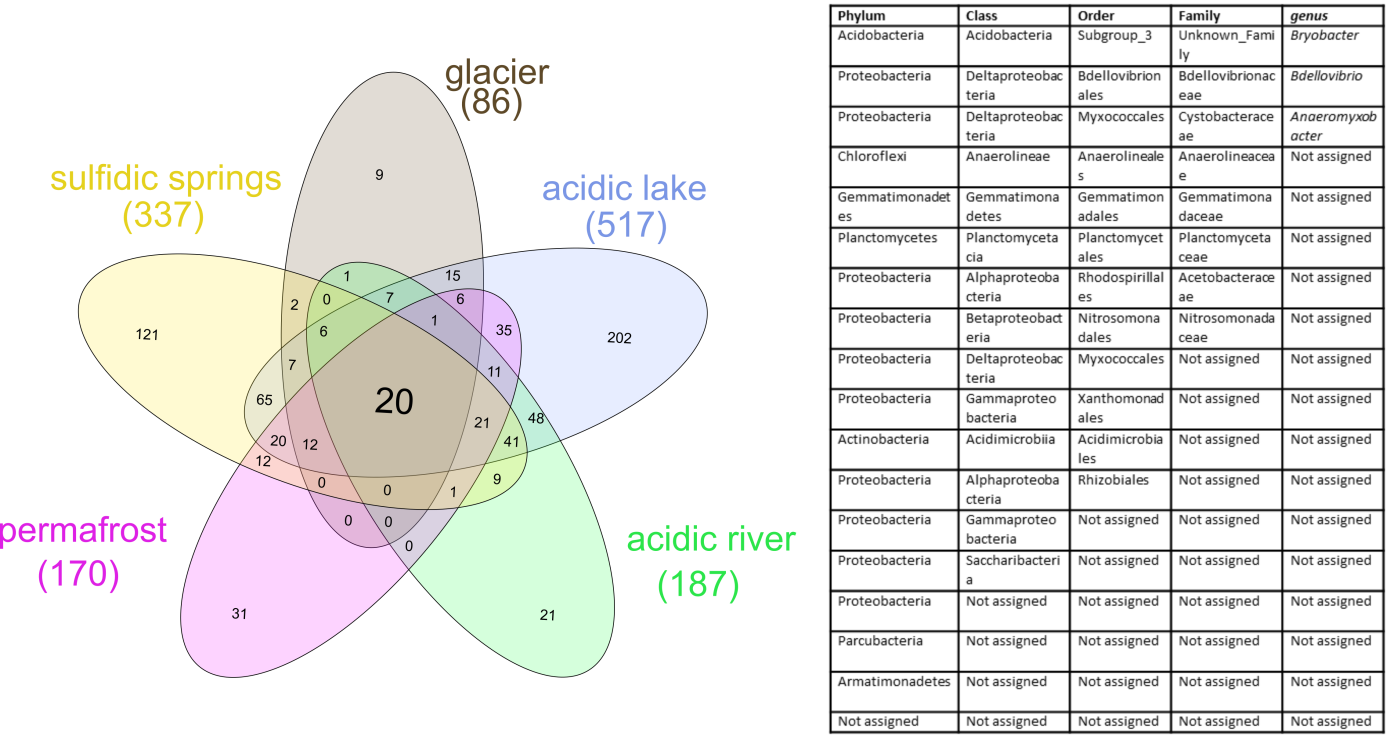


**Supplementary Figure 10: Phylum-level, taxonomic information derived from the metagenomic dataset.** Barchart: all retrieved taxa. Heatmap: Prokaryotic phyla only.


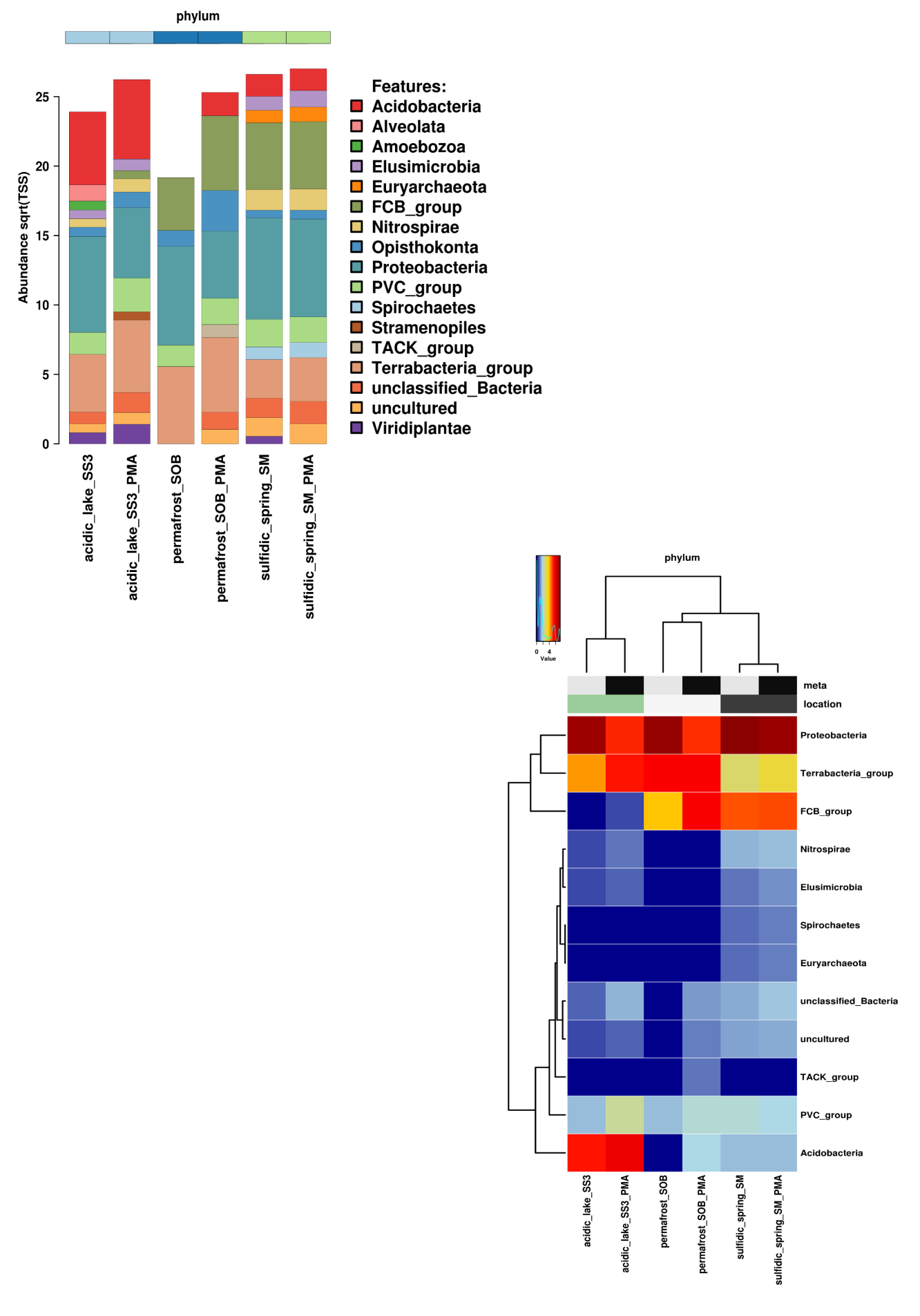


**Supplementary Figure S11: Predicted KEGG3 pathways (Tax4fun) and network of functional genes of the MASE site microbiomes.**

In addition to metagenomic analysis, the computational tool Tax4Fun[18] was applied to predict the functional gene content of the viable microbial community in all sampling sites (here, only PMA treated samples and the PMA untreated hypersaline samples were included). Tax4Fun calculated an estimated relative abundance of KEGG orthology groups (KOG) associated with each sample depending on matches of the representative sequence from each RSV to KEGG organisms. Subsequently, some pathways and genes were manually selected for detailed analyses, based on their relevance for the astrobiological question; the according heatmaps were generated using the R package pheatmap and the network was generated using Cytoscape 2.8.3 [19].

**Results:** We predicted the entire set of present pathways (284) and genes (6,600) for PMA treated samples (including the “hypersaline environment”). Genes encoding functions involved in the basic stress and adaptation system (i.e. the two-component system) were predicted for all biotopes and revealed a general high abundance. This was also true for genes belonging to the family of ABC-transporters. Other stress response systems, such as DNA repair (e.g. homologous recombination, mismatch repair, base excision repair) could be predicted as well for all environments. Several predicted genes with capacity for stress response, sporulation, response to starvation, heat shock, hypoxia etc. were identified, but they differed in their abundance. The most abundant predicted genes (>0.05 % of selected, stress-relevant predicted genes) comprised genes encoding for sporulation, motility, proton pumps, and DNA repair (e.g. *RadA*, *RecN*). Moreover, microorganisms thriving in MASE sites were found to potentially exhibit a wide variety of genes enabling the degradation of diverse chemical compounds ranging from toluene, naphthalene, styrene, glycan and benzoate. Also, genes involved in the degradation of amino acids (in particular, valine, leucine, isoleucine, and lysine) could be predicted. Their abundance was predicted to be the highest in permafrost samples, the hypersaline environment and the acidic environment.

The heatmaps in (a-d) display the relative abundance of predicted pathways in each sample. (a) Shows the genes involved in various synthesis pathways, (b) genes encoding for transporters and repair systems (amongst others), (c) genes encoding for the metabolic pathways, and (d) genes encoding for degradation pathways. In (e) and (f), genes were selected individually and their abundance is shown as networks. Each node represents the summed relative abundance of a certain gene throughout all samples. The edges display the connection to corresponding sampling sites (more clearly displayed in the close-up). For (f), genes with a relative abundance >5 % were selected to generate an overall network.


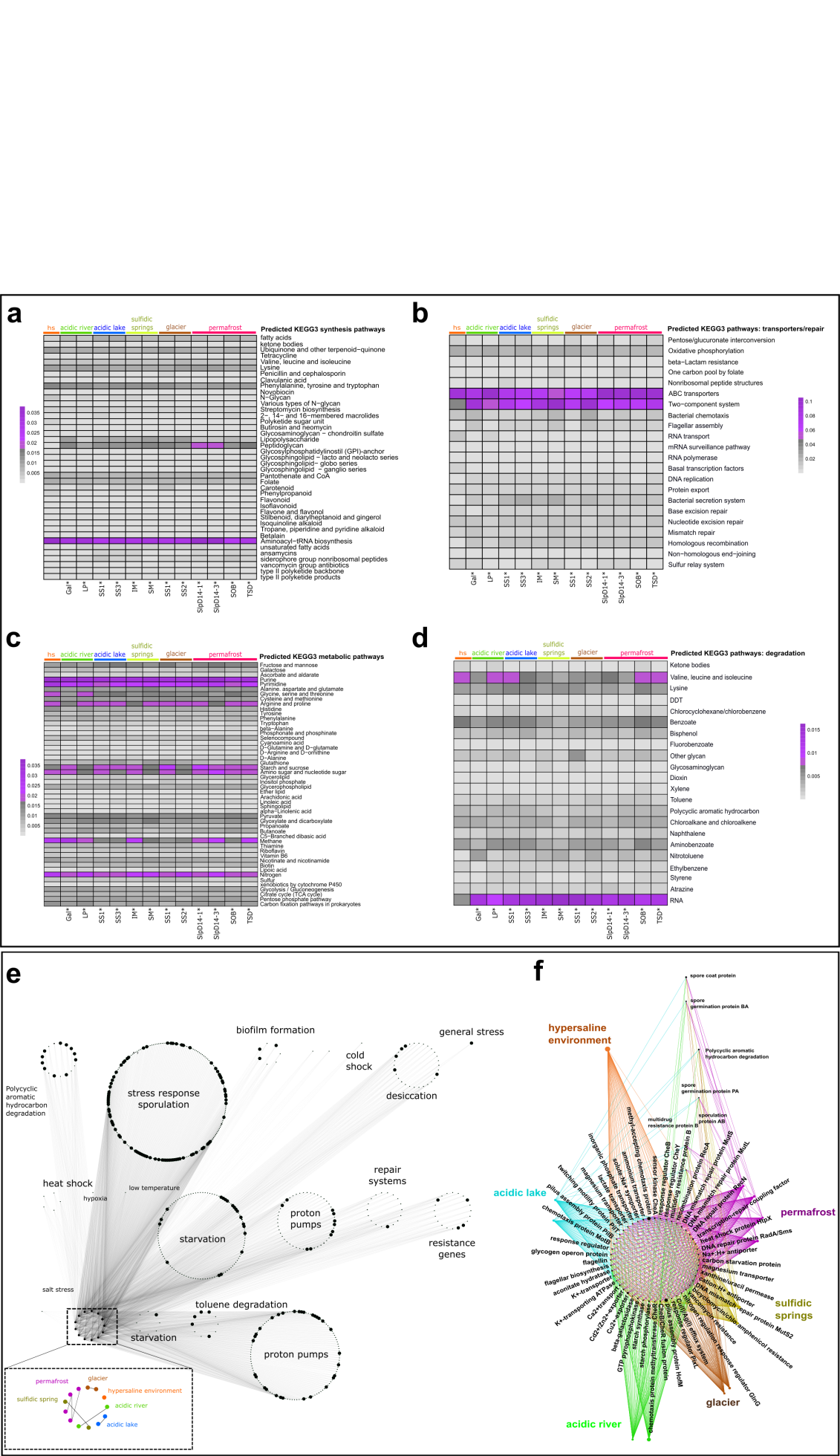


**Supplementary Figure 12: Comparison of metabolic pathways for the core microbiome and the cultivated microorganisms.** For calculation, the RSVs of the viable (excluding the hypersaline environment) were taken into account. The pathways are grouped into pathways involved in Carbon cycle, Amino acid metabolism, fatty acid and lipid metabolism and “others”.


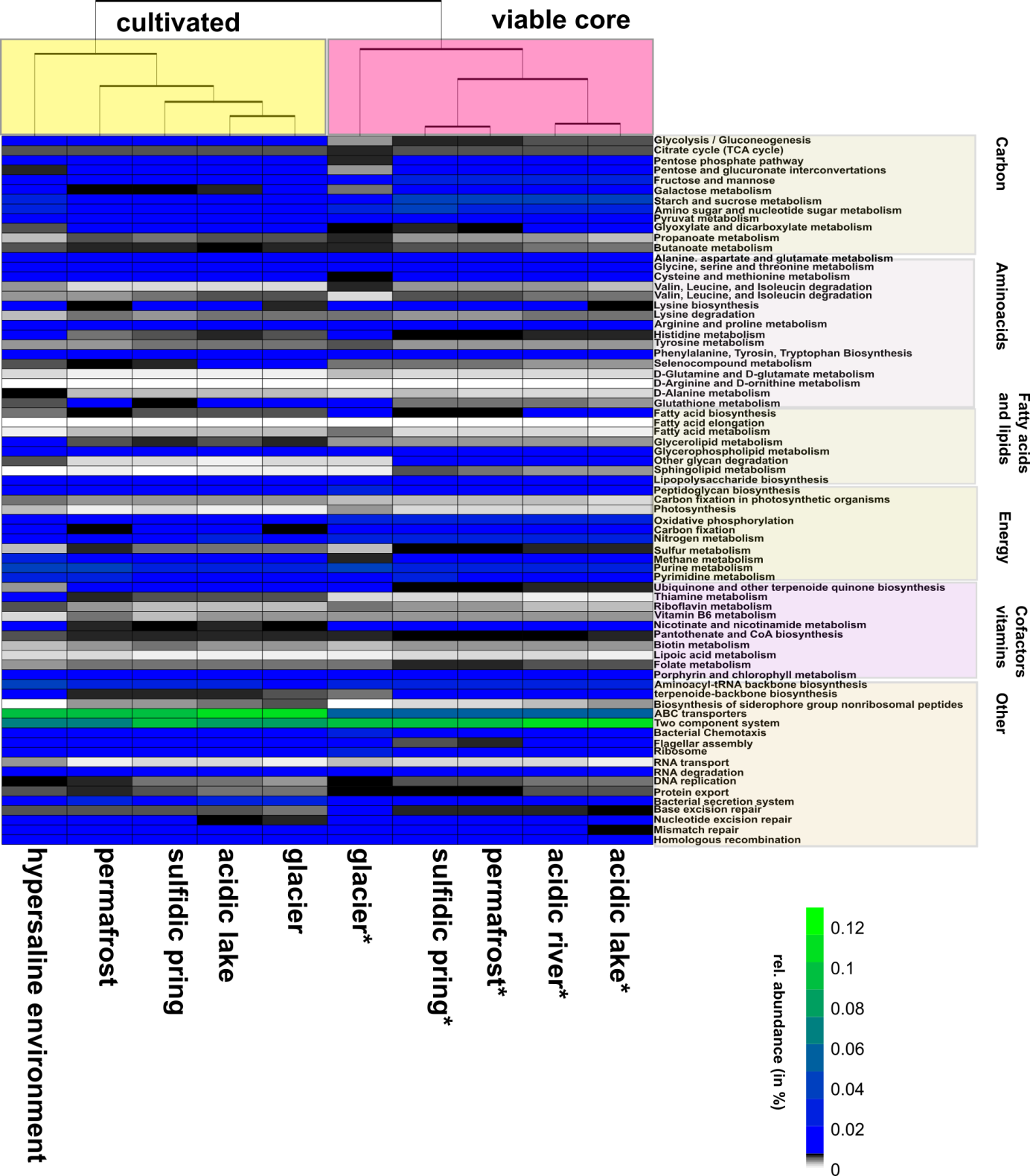


**Supplementary Figure 13: Total organic carbon in MASE samples**, in %. The TOC was analysed using the high-temperature TOC analyser of Shimadzu, ASI-V.

| Sample | Total organic carbon (TOC),  in % |
| --- | --- |
| Acidic lake | 0.12 |
| Sulfidic spring (SM) | 4.87 |
| Sulfidic spring (IM) | 4.84 |
| Permafrost SLpD14-1 | 5.89 |
| Permafrost SLpD14-3 | 1.58 |
| Permafrost TSD | 2.24 |
| Permafrost SOB | 22.7 |

1. Cockell CS, Schwendner P, Perras A, Rettberg P, Beblo-Vranesevic K, Bohmeier M, et al. Anaerobic microorganisms in astrobiological analogue environments: from field site to culture collection. Int J Astrobiol. Cambridge University Press; 2017;1–15.

2. Huber R, Burggraf S, Mayer T, Barns SM, Rossnagel P, Stetter KO. Isolation of a hyperthermophilic archaeum predicted by in situ RNA analysis. Nature. Nature Publishing Group; 1995;376:57–8.

3. Fröhlich J, König H. New techniques for isolation of single prokaryotic cells. FEMS Microbiol Rev. Blackwell Publishing Ltd Oxford, UK; 2000;24:567–72.

4. Kim O-S, Cho Y-J, Lee K, Yoon S-H, Kim M, Na H, et al. Introducing EzTaxon-e: a prokaryotic 16S rRNA gene sequence database with phylotypes that represent uncultured species. Int J Syst Evol Microbiol. Microbiology Society; 2012;62:716–21.

5. Quast C, Pruesse E, Yilmaz P, Gerken J, Schweer T, Yarza P, et al. The SILVA ribosomal RNA gene database project: improved data processing and web-based tools. Nucleic Acids Res. 2013;41:D590–6.

6. Schwendner P, Bohmeier M, Rettberg P, Beblo-Vranesevic K, Gaboyer F, Moissl-Eichinger C, et al. Beyond chloride brines: Variable metabolomic responses in the anaerobic organism Yersinia intermedia MASE-LG-1 to NaCl and MgSO<inf>4</inf>at identical water activity. Front Microbiol. 2018;9.

7. Beblo-Vranesevic K, Rabbow E, Bohmeier M, Rettberg P, Gómez F, Garcia-Descalzo L, et al. Lack of correlation of desiccation and radiation tolerance in microorganisms from diverse extreme environments tested under anoxic conditions. FEMS Microbiol Lett [Internet]. 2018;365. Available from: https://dx.doi.org/10.1093/femsle/fny044

8. Vallenet D, Engelen S, Mornico D, Cruveiller S, Fleury L, Lajus A, et al. MicroScope: A platform for microbial genome annotation and comparative genomics. Database. 2009;2009:1–12.

9. Groemer G, Losiak A, Soucek A, Plank C, Zanardini L, Sejkora N, et al. The AMADEE-15 Mars simulation. Acta Astronaut. Elsevier; 2016;129:277–90.

10. Probst AJ, Auerbach AK, Moissl-Eichinger C. Archaea on human skin. PLoS One. 2013;8:e65388.

11. Moissl C, Rudolph C, Huber R. Natural communities of novel archaea and bacteria with a string-of-pearls-like morphology: molecular analysis of the bacterial partners. Appl Environ Microbiol. Am Soc Microbiol; 2002;68:933–7.

12. Wallner G, Steinmetz I, Bitter-Suermann D, Amann R. Combination of rRNA-targeted hybridization probes and immuno-probes for the identification of bacteria by flow cytometry. Syst Appl Microbiol. Elsevier; 1996;19:569–76.

13. Winston PW, Bates DH. Saturated solutions for the control of humidity in biological research. Ecology. Wiley Online Library; 1960;41:232–7.

14. Kumar S, Stecher G, Tamura K. MEGA7: molecular evolutionary genetics analysis version 7.0 for bigger datasets. Mol Biol Evol. Society for Molecular Biology and Evolution; 2016;33:1870–4.

15. Thompson JD, Gibson T, Higgins DG. Multiple sequence alignment using ClustalW and ClustalX. Curr Protoc Bioinforma. Wiley Online Library; 2002;2–3.

16. Letunic I, Bork P. Interactive Tree Of Life (iTOL): an online tool for phylogenetic tree display and annotation. Bioinformatics. 2006/10/20. 2007;23:127–8.

17. Heberle H, Meirelles GV, da Silva FR, Telles GP, Minghim R. InteractiVenn: a web-based tool for the analysis of sets through Venn diagrams. BMC Bioinformatics. BioMed Central; 2015;16:1.

18. Aßhauer KP, Wemheuer B, Daniel R, Meinicke P. Tax4Fun: predicting functional profiles from metagenomic 16S rRNA data. Bioinformatics. Oxford Univ Press; 2015;31:2882–4.

19. Shannon P, Markiel A, Ozier O, Baliga NS, Wang JT, Ramage D, et al. Cytoscape: a software environment for integrated models of biomolecular interaction networks. Genome Res. Cold Spring Harbor Lab; 2003;13:2498–504.
